# A Self-Directed Method for Cell-Type Identification and Separation of Gene Expression Microarrays

**DOI:** 10.1371/journal.pcbi.1003189

**Published:** 2013-08-22

**Authors:** Neta S. Zuckerman, Yair Noam, Andrea J. Goldsmith, Peter P. Lee

**Affiliations:** 1 Department of Cancer Immunotherapeutics and Tumor Immunology, City of Hope and Beckman Research Institute, Duarte, California, United States of America; 2 Department of Electrical Engineering, Stanford University, Stanford, California, United States of America; National Center for Biotechnology Information (NCBI), United States of America

## Abstract

Gene expression analysis is generally performed on heterogeneous tissue samples consisting of multiple cell types. Current methods developed to separate heterogeneous gene expression rely on prior knowledge of the cell-type composition and/or signatures - these are not available in most public datasets. We present a novel method to identify the cell-type composition, signatures and proportions per sample without need for a-priori information. The method was successfully tested on controlled and semi-controlled datasets and performed as accurately as current methods that do require additional information. As such, this method enables the analysis of cell-type specific gene expression using existing large pools of publically available microarray datasets.

## Author Summary

Gene expression microarrays are widely used to uncover biological insights. Most microarray experiments profile whole tissues containing mixtures of multiple cell-types. As such, gene expression differences between samples may be due to different cellular compositions or biological differences, highly limiting the conclusions derived from the analysis. All current approaches to computationally separate the heterogeneous gene expression to individual cell-types require that the identity, relative amount of the cell-types in the tissue or their individual gene expression are known. Publically available microarray-based datasets, which include thousands of patient samples, do not usually measure this information, rendering existing separation methods unusable. We developed a novel approach to estimate the number of cell-types, identities, individual gene expression and relative proportions in heterogeneous tissues with no a-priori information except for an initial estimate of the cell-types in the tissue analyzed and general reference signatures of these cell-types that may be easily obtained from public databases. We successfully applied our method to microarray datasets, yielding highly accurate estimations, which often exceed the performance of separation methods that require prior information. Thus, our method can be accurately applied to any heterogeneous dataset, where re-examination and analysis of the individual cell-types in the heterogeneous tissue can aid in discovering new aspects regarding these diseases.

This is a *PLOS Computational Biology* Methods article.

## Introduction

Gene-expression profiling of whole tissues is affected by the different cell types that exist in the tissue and their relative proportions. Thus, changes detected by differential expression analysis may reflect differences in the proportions of the cell-types between samples rather than an important mechanistic change in gene-expression. For example, the proportion of tumor cells in breast cancer biopsies were found to significantly affect expression profiles, where consideration of these proportions improved response prediction [1]. Therefore, profiling of heterogeneous tissues rather than sorted cell-types can greatly limit the conclusions derived from such analyses.

Solutions for experimentally separating cell-types from heterogeneous tissues include laser-capture microdissection to isolate morphologically distinguishable cells and flow cell sorting to purify cell-types from a tissue. However, in addition to the time-consuming nature of these methods, they may result in insufficient quantities of RNA, where amplification steps may introduce artifacts to the gene expression data [2]. Single cell RNA sequencing is becoming feasible; however, experimental costs are high and few studies utilize this method on a large patient pool. To address this issue, several approaches to computationally separate expression profiles of heterogeneous tissues into their constituent cell-types along with their relative proportions per sample have been developed. Most approaches utilize a linear model that has been demonstrated to yield accurate expression estimates [3], [4]; in this model the gene-expressions of each cell-type are added up to form a mixed expression, where each cell-type is weighted according to its relative proportion in the tissue.

All currently existing separation methods require some a-priori information about the tissue analyzed, such as the number of cell-types and their relative proportions in the tissue [3], [5], or the number of cell-types, their identity and their purified gene expression [4], [6]–[8], or just the number of cell-types in the tissue [9]–[11]. A preliminary attempt to estimate the number of cell-types in the mixed data, but not their identities, has also been proposed [12]. However, most studies do not purify the different cell populations in the tissue, enumerate their proportions or verify their identity, rendering these methods inapplicable to separation of such heterogeneous gene-expression datasets.

In this study, we have developed a novel approach to blindly separate heterogeneous gene-expression data, i.e., without using any specific prior information regarding the analyzed dataset. In addition to separating the heterogeneous tissue to the individual gene expression profiles of its constituent cell-types and their relative proportions per sample, the algorithm described here performs an extra step of identifying the number of cell-types in the tissue and their identities. Compared to existing methods, the only a-priori information the algorithm requires is an initial guess of the cell-types that may exist in the analyzed tissue and purified reference signatures of these cell-types, which may be found in abundance in publically available databases. We have successfully tested our algorithm on three publically available databases in which all the conditions are controlled and on a publically available semi-controlled dataset with estimated cell-type proportions.

To our knowledge, this method is the first that can practically be applied, in a “plug and play” fashion, to any existing dataset of heterogeneous tissue samples, in order to identify the cell-types in the samples, their identities, their proportions per sample and their separated gene-expression signatures without requiring any prior knowledge.

## Results

### A novel approach to blindly identify and separate the cell-types in heterogeneous tissues

The proposed algorithm is based on a hyper-spectral imaging method developed by Piper *et al.* [13]. It is designed to identify the number of cell-types in heterogeneous tissue samples, their identities, their relative proportions per sample and separate their individual gene expression signatures. The proposed algorithm includes three parts (see methods section for more details). In the first part, non-negative matrix factorization [13] is used to obtain an initial estimate of expression profiles for each cell-type. A rough initial estimate of the numbers and identities of the cell-types in the tissue is required. This estimate can include cell-types that may not exist in the tissue. However, if a true cell-type is not included in the initial estimate, then the algorithm will not detect this cell-type and there may be ambiguities in the resulting cell-type signatures and proportions. In addition, purified reference signatures are required for each of the cell-types included in the initial estimate. Such reference signatures may be found in abundance in the Gene Expression Omnibus (GEO) [14] and may be general, i.e., not be disease, tissue, experiment or study-specific. In the second part of the algorithm, the true number of cell-types is estimated using the symmetric Kullback-Leibler divergence (SKLD) between each of the estimated cell-type profiles and the initial cell-type reference signatures, where the closest estimated profiles are then chosen as the final cell-types. SKLD, a measure used to calculate the difference between two probability distributions, is used here as a measure of distance, as we describe in the methods section under (5). In the final part, the cell-type proportions are computed per sample, using the method of non-negative least squares (NNLS), a method that solves matrix equations algebraically with an added constraint for non-negative elements, as we describe in the methods section under equation (3). Additional adjustments, motivated by the application of the algorithm to gene-expression data include: (a) majority voting, where the final identity of the cell-types is chosen from the results of several algorithm runs with random initializations, and (b) usage of classes, where several input reference signatures may be grouped into one “class” of cell-type. These adjustments were added to the algorithm to improve separation capabilities of cell-types with similar signatures and increase the algorithm's robustness to noisy reference signatures (for additional details see the methods section).

### Application of the algorithm to controlled datasets

**Figure 1:**
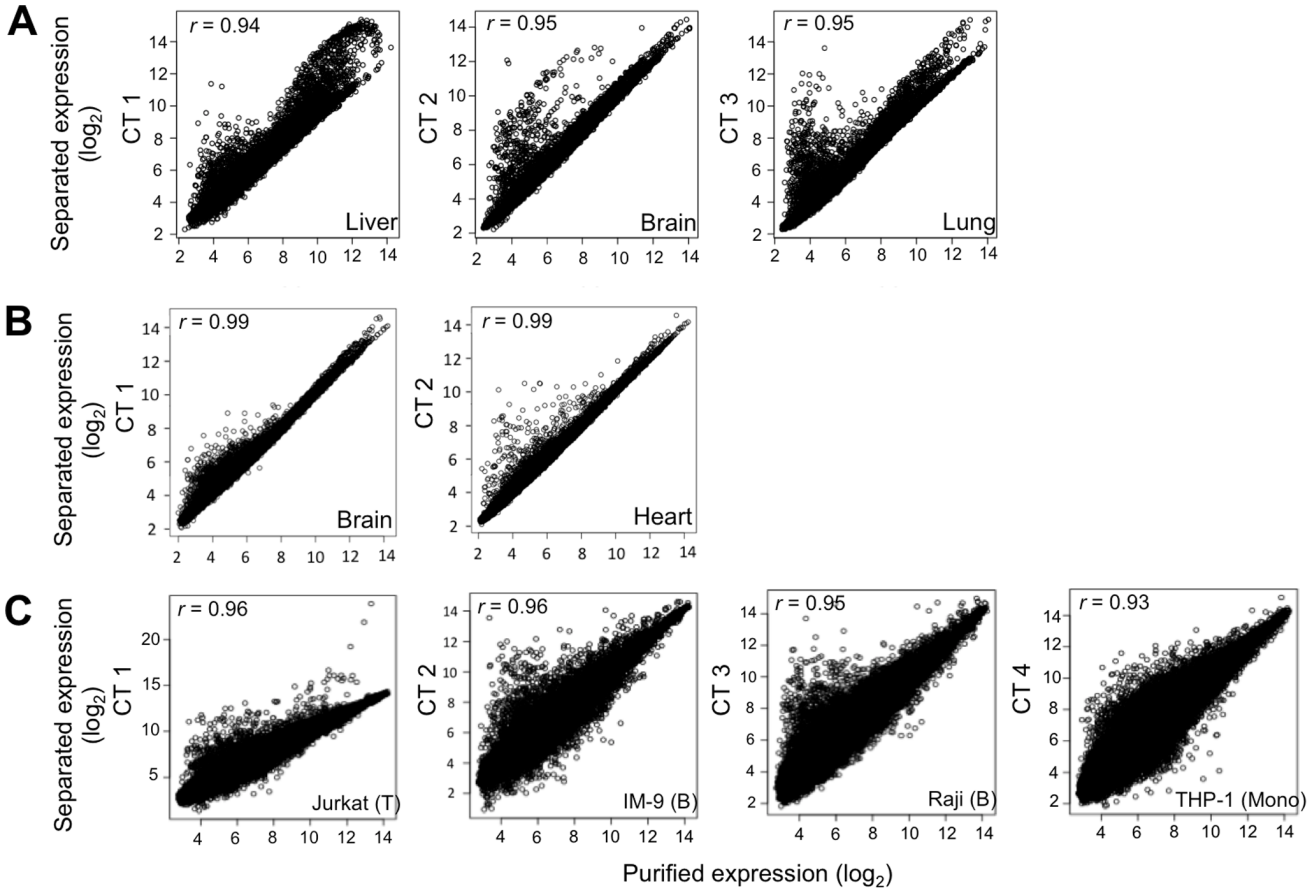
Blind separation yields accurate estimations of separated cell-type gene-expression. Gene expression measurements of each separated cell-type plotted against the gene expression of its corresponding purified cell type from the same study, in (**A**) liver-brain-lung dataset [3], (**B**) heart-brain dataset [15], and (**C**) T-B-Monocytes dataset [4]. Correlation coefficients between the separated and purified gene expressions are denoted for each cell type. CT = cell-type, r = correlation coefficient.

**Figure 2:**
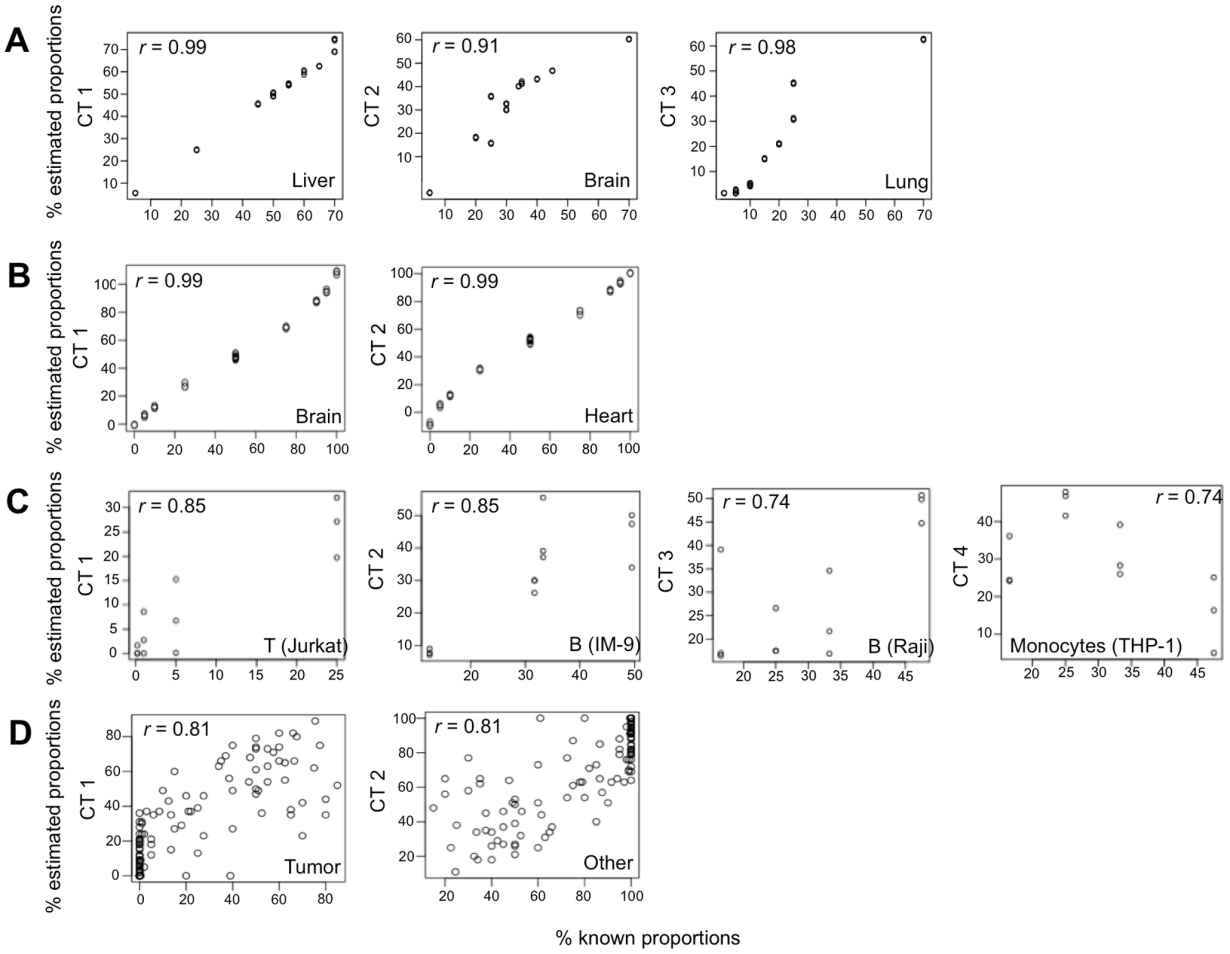
Blind separation yields accurate estimations of separated cell-type proportions. Known cell-type proportions plotted against the estimated cell-type proportions in (**A**) liver-brain-lung dataset [3], (**B**) heart-brain dataset [15], (**C**) T-B-Monocytes dataset [4], and (**D**) prostate cancer dataset [7]. Correlation coefficients between the known and estimated proportions are denoted for each cell-type.

**Figure 3:**
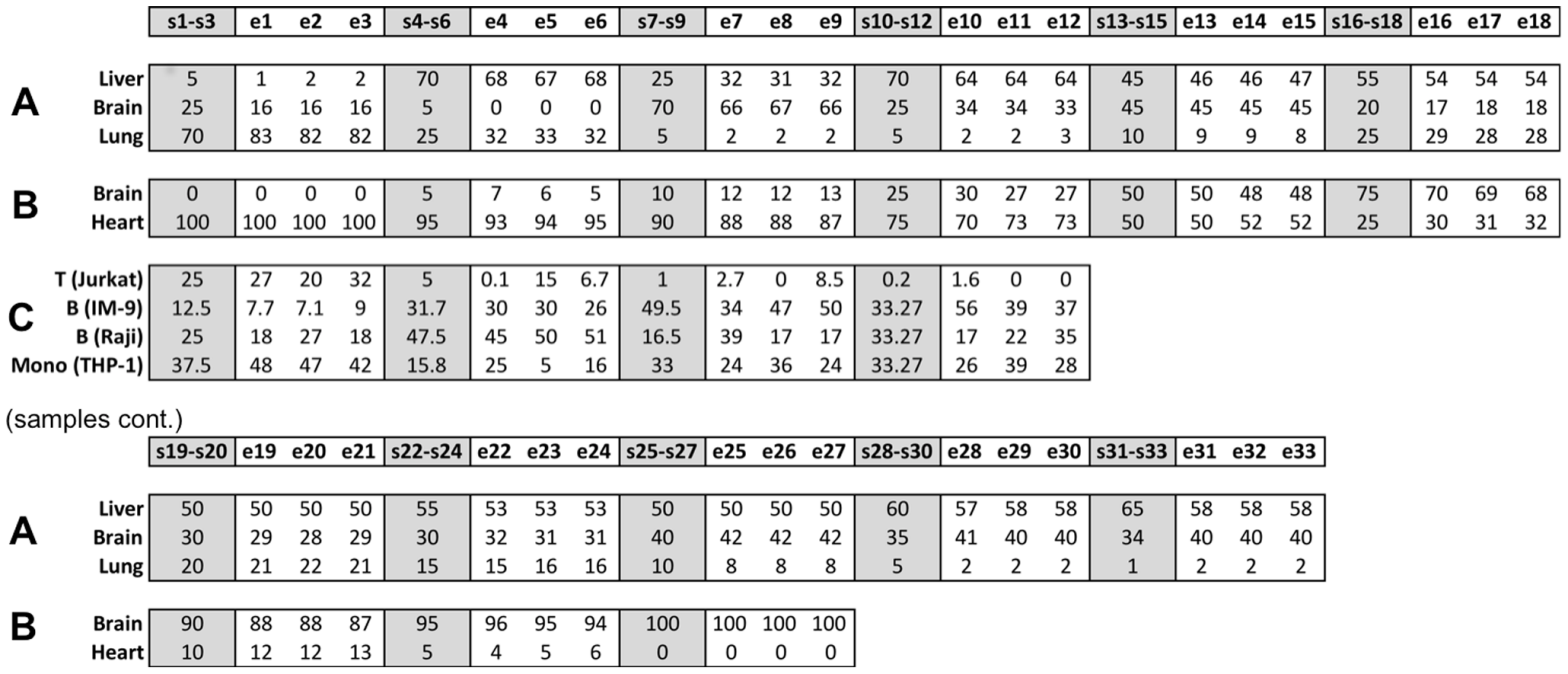
Blind separation yields accurate estimates of cell-type proportions per sample. Comparisons between the known cell-type proportions and the estimated proportions per-sample for cell-types in the controlled datasets. (**A**) The liver-brain-lung dataset [3] included 33 samples, (B) The heart-brain dataset [15] included 27 samples, and (**C**) The T-B-Monocytes dataset [4] included 12 samples. Known proportions are shown in the grey columns, denoted by “s” and were measured as triplicates; estimates yielded by the blind separation algorithm are shown in the white columns and are denoted by “e”.

We tested the algorithm on three publically available datasets in which known proportions of known cell types were mixed and their gene-expression was measured. The liver-brain-lung dataset includes samples of rat liver, brain and lung cell mixtures [3]. The purified cell-type reference signatures were collected from GEO and included rat liver, brain, lung, intestine, heart and granulosa cell gene-expression profiles from different studies (see “microarray data” in methods section; Figure S1A). Although the mixed samples included only three cell-types, more than three cell-types were inputted to the algorithm as the initial number of cell-types to test the algorithm's ability to discern the correct number. The algorithm successfully identified three cell-types in the mixed samples and their correct identities, i.e., liver, brain and lung. High correlations were found between the gene-expression profiles of each estimated cell-type to the profile of its corresponding purified cell-type taken from the same study (Figure 1A), in addition to shortest SKLD distances (Figure S1B). These correlations, obtained by our blind separation method, were in the range of the correlations reported in the Shen Orr *et al.* study where the number of cell-types, their identities and their proportions per sample were input to the algorithm, and even higher in the case of the lung cell-type [3]. High correlations were also obtained between the actual and estimated cell-type proportions (Figure 2A), in addition to shortest SKLD distances (Figure S1C). Sample-by-sample comparison of the estimated proportions of each cell-type shows that our algorithm is successful in reconstructing accurate proportions per cell-type per sample, with an average absolute error of 3.4%±2.3 (Figure 3A). In addition, the resulting expression signatures had shorter SKLD distances and thus were closer to the original purified expression profiles compared to the input profiles, demonstrating that the algorithm successfully advanced the input signatures (Figure S1D). Note that we use SKLD distances as the distance measure in results testing, as it is the measure used in the algorithm itself.

The Heart-Brain dataset includes samples of heart and brain human cell mixtures [15]. Purified cell reference signatures were collected from GEO and included myocardial (heart) cells, brain cells from the entorhinal cortex and grey matter, oocytes and hepatocytes from different studies (see “microarray data” in methods section; Figure S2A). We unified the two heart signatures obtained from different studies under the class “heart” and the two brain signatures obtained from different brain tissues under the class “brain”. The algorithm successfully identified the true cell-types, i.e., heart and brain. The cortex brain cell-type was identified in all algorithm majority voting runs whereas the brain grey-matter cell-type was identified in only 20% of the majority voting runs, suggesting that the cells in the mixtures are most probably cortex cells or cells with a similar signature. The estimated cell-type expression profiles showed the highest correlations (Figure 1B) and shortest SKLD distances (Figure S2B) to their corresponding purified cell-types taken from the same study. High correlations (Figure 2B) and shortest SKLD distances (Figure S2C) between the estimated and known cell-type proportions were obtained, with a low average absolute error of 1.7%±1.85 (Figure 3B). Finally, the resulting expression signatures were closer to the original purified expression profiles compared to the input profiles (Figure S2D).

To test separation of cell-types with similar signatures, we chose the T-B-Monocytes dataset, containing mixtures of T, Monocyte and two types of B cell lines [4]. Purified cell reference signatures collected from GEO included human immune cell lines of T-cells, B-cells, Monocytes, NK cells and epithelial cells (see “microarray data” in methods section; Figure S3A). The algorithm successfully identified all three cell-types (T, B, Monocytes) and also successfully discerned between the two types of B cell-lines, yielding a total of four resulting cell-types – T Jurkat, B Raji, B IM-9 and Monocyte THP-1 cell lines. High correlations were obtained between the gene-expression profiles of each estimated cell-type to the profile of its corresponding purified cell-type taken from the same study (Figure 1C) and between the estimated and known cell-type proportions (Figure 2C), in addition to shortest SKLD distances (Figures S3B–C). The average error in cell-type proportions per-sample obtained by our blind separation method was 5.7%±3.3 (Figure 3C), which is close to the error reported by the original study separating these same samples, where the number of cell-types, their identity and their gene expression profiles were given as an input [4]. In addition, the resulting expression signatures were closer to the original purified expression profiles compared to the input profiles (Figure S3D). A lower average error in cell-type proportions per sample (3.67%±3.04) and higher correlations between the estimated and known cell-type specific gene-expression profiles were obtained in an algorithm run where the two B cell-line types were unified under the “B cells” class (Figures S3E–I). In this run, where the goal was to separate between the different immune cell-types in the mixed samples, the algorithm successfully identified the three cell-types – T-cells, B-cells and Monocytes.

### Application of the algorithm to a semi-controlled dataset

We tested the algorithm on a semi-controlled dataset of prostate cancer in which cell-type proportions were estimated by a pathologist [7]. The cell-types in the analyzed tissue were carcinoma, benign (BPHE) and dilated (DCAE) epithelial and stromal cells. Purified cell signatures of prostate tumor cell lines, benign prostate cells, normal prostate epithelial cells, stroma surrounding invasive prostate tumors and normal stroma were collected from GEO (see “microarray data” in methods section; Figure S4A). Data were available for the percentage of tumor cells in each sample, thus classes unifying the prostate cell-lines and the other cell-types under “tumor” and “other”, respectively, were used. High correlations were obtained between the pathologist's estimated cell-type proportions and the cell-type proportions estimated by the algorithm (Figure 2D), with an average error per sample of 12.44%±12.41 (Figures S4B–C). Compared to the results obtained in the controlled datasets, the cell-type proportions prediction error was higher in this case. This could be due to the fact that no specific signatures for BPHE and DCAE cells were found within the same/similar microarray platforms. Therefore, general signatures for stromal and epithelial cells were used, which may have decreased the prediction accuracy. However, it is more likely that the pathologist's estimations of cell-type proportions were not fully accurate. Indeed, the lowest error calculated between the actual and estimated cell-type proportions in that study was: 9.5% and 12.5% for tumor and stromal cells, respectively [7], where our blind separation algorithm reached similar error rates with non-specific signatures.

## Discussion

Gene-expression analysis of whole tissues, which are heterogeneous in nature and consist of a mixture of several cell-types, are utilized extensively and are highly abundant in public repositories such as GEO [14]. However, it is now becoming clear that the identity, composition and profiles of individual cell-types are extremely important to the process of unraveling the biology of each cell-type population and the interplay between the populations in both healthy and disease states. Due to the expense and difficulties of separating them, only a limited amount of studies profile and analyze individual cell-types. More importantly, public repositories are replete with existing data of whole tissues including thousands of patients, treatments, tissues and cell-types. This rich trove of data is from experiments that may never be repeated using such large patient pool or experimental conditions. Our techniques can realize the great potential of these data, which contains much information about the constituent individual cell-types in heterogeneous tissues that, to date, have not been fully interrogated.

Computational methods have been developed to allow the separation of heterogeneous tissues into their cell-type constituent profiles and/or relative proportions [3]–[12]. However, all currently existing separation methods require that the number of cell-types in the tissue, their identity, or their relative proportions in the analyzed tissue are known. Such information rarely exists, as most profiling studies do not purify the cell-types in the tissue, extract their proportions or verify their identity, rendering the existing separation methods non-usable for most existing datasets; Rather, these datasets are usable only in experiments designed in advance to allow for the separation technique.

We have developed a separation method that requires no a-priori information about the tissue analyzed other than an initial rough estimate of the cell-types that may exist in the tissue samples analyzed. This is a reasonable input to ask for and relatively easy to find, as information regarding the composition of most tissues is readily available in the literature and public databases such as GEO are replete with many types of purified cell-types from various experiments. As our algorithm does not require the purified cell-type profiles to be disease, tissue or even study-specific, one can simply use any relevant purified profile as an input to the algorithm. These properties render our algorithm the only useful method to separate most publically available heterogeneous microarray datasets.

We successfully applied our separation technique to three controlled datasets with known proportions and cell types [3], [4], [15] in addition to a semi-controlled dataset where cell-type proportions per sample were estimated by a pathologist [7], to test the method on a dataset that resembles the heterogeneous datasets available in the literature rather than on datasets specifically engineered for separation. Our blind separation technique accurately extracted the relative cell-type proportions per sample and their separated gene-expression signatures and performed just as well, and in some cell-types even better, than other reported separation techniques that require different types of input information about the dataset analyzed to be available. Most importantly, our technique successfully identified the number of cell-types in the tissues analyzed and their identities. These features are not included in any of the reported separation techniques, and are in fact considered as an integral input for the usage of these techniques. It is these features that are mostly unavailable for publically available datasets, or any dataset in which they have not been experimentally identified. In addition, the cell-type populations and proportions in a tissue are not always consistent amongst different individuals, which renders the identification of those populations and their identities crucial.

The algorithm's robustness to varying input signatures was demonstrated by using additional cell-type signatures that were not related to the analyzed tissue as input to each controlled dataset (e.g. the intestine, heart and granulosa cell-types were input to the liver-brain-lung dataset). To address the algorithm's robustness to signatures of different qualities, signatures from different studies were used for the same cell-type and gathered under the same class (e.g two T-cell Jurkat and B-cell Raji cell-lines from different studies were input to the T-B-Monocytes dataset). The algorithm identified the correct number of cell-types and their correct identities in all examples. In general, the algorithm performed better when separating cell-types that were very different from one another as in the heart-brain dataset, compared to cell-types that were very similar to each other such as in the T-B-Monocyte dataset. However, in the latter example, given that no a-priori data about the mixed tissue was provided, the algorithm still yielded accurate results. In particular, the algorithm identified all three cell-types (T, B and Monocytes) with an error that was close to that reported by the original study where the number of cell-types, their identity and their true gene-expression profiles were given as an input [4]. Moreover, the algorithm also successfully separated the two B cell-lines, cell-types with an almost identical gene expression. A comparison between the true purified signatures from the same study to the input signatures mined from GEO and the resulting signatures inferred by our algorithm showed that, in each of the datasets explored, the resulting signatures were always closer to the true signatures than the signatures from GEO, demonstrating that our algorithm successfully identifies the input signatures close to the true ones.

Compared to existing algorithms, our algorithm yielded at least comparable results, and in some cases better results (such as predicting the lung cell-type in the liver-brain-lung dataset). An important distinction is that our algorithm does not require the a-priori information required in existing algorithms and, in contrast with those algorithms, it is able to determine the number of cell-types in the heterogeneous tissue and their identities. To demonstrate the importance of this added capability, we compared the performance of our algorithm to an NNMF approach, without the cell-type determination step, which is initialized in the same manner as our algorithm (Figures S5, S6). We also compared our algorithm's performance to that of a simple NNLS-based algorithm, used here as a bench-mark due to the fact that most existing algorithms are based on NNLS [3]–[12], [16] (Figure S7, S8). In both cases, even a small error in the guess of the number of cell-types (e.g. guessing 4 cell-types instead of 3) deteriorates the performance of these existing algorithms, demonstrating that the cell-type determination step is crucial for good separation. This emphasizes the usefulness of our algorithm not only in situations where no a-priori information exists, but also in the more common scenarios where one has a good but not perfect guess of the cell composition with an error of at most one or two cell-types.

In summary, our blind separation technique successfully identifies the cell-type composition in heterogeneous gene-expression data, and provides high-accuracy estimates of cell-type specific signatures and their relative proportions per sample. The only information the algorithm requires is an initial estimate of the cell-types that may exist in the tissue analyzed and their signatures, which can be easily found in public databases such as GEO. This method is especially advantageous for re-analyzing existing microarray data for which no additional information is available, allowing re-examination and extraction of information for individual cell-type populations while taking advantage of already-existing, large-scale microarray datasets.

## Methods

### Linear model for separation of gene-expression

The following linear model is widely used for separation of gene expression [3]–[12], [16]:
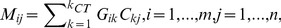
(1)Where *M*_ij_ is the mixed expression matrix of gene *i* in sample j, 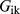 is the separated cell-type specific gene-expression matrix of gene i in cell type *k* and 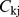 is the matrix of relative proportion of cell type *k* in sample *j*; 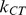 is the total number of cell-types in the tissue, *m* and *n* are the total number of genes and samples, respectively [9]. Studies based on the model in (1) have shown that separation of mixed data with known proportions yielded cell-type specific expression estimates that were highly correlated with the corresponding purified cell gene-expression [3], [4], rendering the linearity assumption acceptable. All currently existing approaches, whether they use the linear model or not, require some a-priori information about the tissue analyzed, such as the number of cell types, their identity or their relative proportions in each sample [3]–[12], [16]. In this work, we are interested in estimating **G** and **C**, from the observation **M**, without explicit a-priory knowledge of the number of cell-types in the tissue, 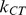, or their identities (note that we will use upper-case boldface letters to denote matrices and lower-case boldface letters to denote vectors). Rather, we consider a collection of 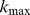 cell-types representing all possible cell-types assumed to comprise the analyzed tissue. This is a hypothesis-testing problem, where each possible combination of cell-types is a hypothesis. Our objective is to choose the correct hypothesis, i.e., to determine which cell-types exist in the analyzed tissue. Assume that *T* is a label of a cell-type. We begin with a collection of labels 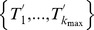 that contains the true composition of cell-types. Specifically, if the true composition of cell-types in a given tissue sample are labeled by 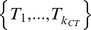, we require that for each 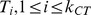, there exists 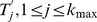, such that 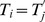. This is a reasonable assumption from a biological point of view, since if the tissue type is known then in most cases the cell-types that may exist in that tissue are also known. Note that if the initial collection of 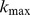 cell-types does not include one of the true cell-types, then this specific cell-type, its expression signature and relative proportions per sample will not be detected by the algorithm and there may be ambiguities in the resulting cell-type signatures and proportions. After estimating the true hypothesis, we estimate the parameters under that hypothesis, i.e. the specific cell-type expression (**G**) and the relative proportion of each cell-type per sample (**C**). The algorithm that we propose requires as input purified gene-expression reference signatures **l** *_i_* for each cell-type label 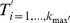, where 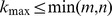. The latter constraint is necessary to have a unique solution to (1), i.e. unique matrices **G** and **C**, up to normalization and permutation, which satisfies the decomposition in (1). These reference signatures need not be identical to the purified signatures that comprise the original columns of the matrix **G** but only need to be taken from the same cell-type. Note that these reference signatures may be acquired from a different experiment, lab or tissue and are found in abundance in gene expression repositories such as GEO [14].

### The relation to hyper spectral Imaging

Separation of gene-expression can be viewed as a special case of a more general class of problems known as Nonnegative Matrix Factorization (NMF) problems, defined as follows: given a nonnegative data matrix M, find the smallest dimension matrices G and C with non-negative entries such that
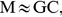
(2)where G is referred to as an end-members matrix (where end-members are classes of composing materials that make up the object M [13]), and C represents the relative proportions in which the end-members are mixed in M i.e, G's ith column represents the signature of the ith end-member, and C's kth entry represent the relative proportion of the kth end-member in the jth data vector 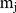. This is equivalent to writing (1) in a matrix form, where each data vector 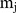 represents microarray measurements of sample j. Each cell-type is an end-member, where G's ith column represents the gene signature of the ith cell-type. The jth column of C represents the relative proportions of the cell-types (whose signatures comprise the columns of G) in sample j. If the number of cell-types is smaller than the number of samples, the dimensions of G and C are smaller than the dimension of M, and the problem in (1) is a special case of the problem in (2).

The algorithm proposed in this paper is an adaptation of an NMF algorithm by Piper et al. [13] that was originally designed for spectral analysis of space objects. Piper et al. studied the problem of identification and classification of space objects whose orbits are significantly distant (e.g., geosynchronous satellites) or whose dimensions are small (e.g., nanosatellites) from ground-based telescope spectral measurements. In their problem, an object is classified by determining the characteristics of the material that make up its spectral trace. Each data vector m_j_ represents a spectral trace (i.e. the spectral image) of the jth object. G's ith column represents the spectral signature of the ith material in the object (end-member). Piper et al.'s hyper-spectral analysis approach is useful for analysis of gene-expression microarrays due to the use of prior knowledge. Their method uses a stored set of laboratory-obtained spectral signatures of space object materials obtained in a different experiment to determine the number of end-members. These stored signatures are not necessarily identical to the underline signatures but are only close to them. This approach is very appealing for separation of gene-expression, since in most cases the purified cell-types are not separated and profiled separately in the same experiment. Furthermore, it is possible to obtain cell-type specific reference signatures and use them for any analysis involving similar cell types. Despite the similarity between the two NMF applications, i.e. gene-expression analysis and spectral analysis, extensions to Piper et al.'s algorithm designed for spectral analysis were needed for the gene-expression analysis, as described in the following section.

### Algorithm

The proposed algorithm includes three major parts. In the first part, we obtain an initial estimate of the matrix **G** using 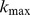 as the number of columns. In the second part we estimate the true number of cell-types, 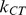, their identities, and the cell-type expression signatures matrix **G**. In the final part we compute the cell-type proportions matrix **C**. A detailed description of these steps is given in the following.

#### Initialization

The algorithm receives as input: (a) an *m×n* matrix **M**, and (b) an *m×* 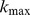 matrix **L**, where **M** is the mixed matrix to be separated with *m* genes and *n* samples and **L** is the reference signatures matrix with *m* genes and 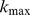 columns. Both **M** and **L** have non-negative entries and are normalized such that each column sums to its mean. The matrices **H** and **W**, which represent intermediate estimates of the **C** and **G** matrices, are initialized as follows. The entries 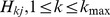, 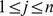 are realized values of independent random variables, uniformly distributed between zero to one. The matrix **W** is initialized with the reference signatures matrix **L** and the columns of **W** are scaled to sum to one.

#### Evaluation of H and W

In the first stage, the algorithm receives the matrix **M** and the integer 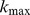 as inputs and outputs **H** and **W** such that
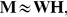
(3)using NMF [13]; i.e., **H**, **W** minimizes 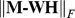 where 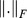 is the Frobenius norm (the root sum of squares of the entries of the matrix), under the constraint that **H** and **W** have positive entries and the columns of **W** sum to one. The matrices **H** and **W** serve as intermediate representations of the matrices **C** and **G**, respectively.

#### Estimation of 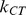 and G

The true number of cell-types in **M**, 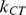 is estimated by:
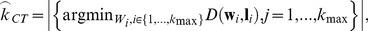
(4)Recall that 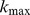 is greater than the true number of cell-types 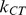, thus some of the columns of the matrix **W** are redundant. Each column in **L**, 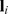, is associated with a column in in **W**, 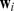, to which it has the minimal distance *D*. Here, SKLD is used, as in Piper *et al.* [13]. The SKLD is defined as follows, let *w* and *d* be two signatures and let 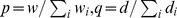, the SKLD defined as
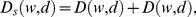
(5) where 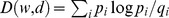. We have also run the algorithm using Euclidean distance and correlation as the distance measures, however the results were not as accurate as using SKLD (not shown). This may be explained by the fact that the SKLD is most suitable with the NMF used in our algorithm, as it only considers arguments with positive values. The estimated number of cell types, 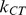, is set to the number of chosen columns in **W**. Note that it is possible that some of the columns in **W** will not be chosen. The cell type identity of each of the chosen 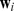 columns is determined according to its corresponding 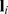 column. In cases where more than one column in **L** is associated with a certain 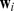, the identity of that 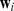 is determined according to the 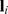 it has the minimal SKLD from. The estimated **G** matrix, 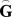, is then constituted from the chosen columns of **W**.

#### Estimation of C

The estimate of the matrix **C** matrix, 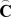, is obtained by using NNLS [3], [9], [13] using 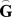 and **M**, such that
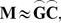
(6)under the constraint that the entries of 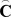 are greater than or equal to zero. Finally, the rows of 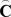 are normalized to 1 to represent cell-type proportions. The output of the algorithm is the matrices 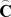 and 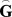, representing the proportions of each cell type in each sample and the specific gene expression for each separated cell type, respectively. Pseudo code of the algorithm is given in Text S1.

#### Majority voting

The NMF algorithm used to evaluate **H** and **W** is not guaranteed to converge to a global minimum [13], as the NMF is not a convex optimization problem. This problem is most significant in cases where the cell-types have similar signatures (e.g. immune cell subsets as in the T-B-Monocytes dataset). To overcome this problem, we have initialized the **W** matrix with the input signatures matrix **L** and, in addition, set an option to run the algorithm several times using random initializations of **H**. Each run yields the estimate 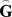 in which each column represents a cell-type that was chosen by the algorithm. The algorithm decides whether a certain cell-type is chosen for the final estimate of **G**, 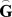, if it is chosen more than a certain threshold, defined as the percentage of the number of times this cell-type was chosen out of the number of total runs. The estimated gene expression of each chosen cell-type is set to the average of the gene-expression of all corresponding estimates of this cell-type in each run it was chosen. The final estimate of the number of cell-types 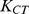 is set to the number of columns of the final 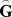 matrix.

#### Classes

The algorithm utilizes the reference signatures matrix **L**. As such, it is sensitive to the signatures provided by the user and may fail to accurately separate cell-types in cases where the cell-types are very similar or if the user is missing a-priori information regarding the tissue to be separated, e.g. the exact nature of the cell type, tissue or experimental conditions. To improve performance in such cases, we have allowed for reference signatures to be grouped into classes with a single label. For example, to separate colorectal tumor cells of an unknown subtype from a mixed tissue, reference signatures for several colorectal tumor types representing different tumor subtypes may be provided and will constitute the class “colorectal tumor”. An additional example for using classes includes unifying several signatures for one cell type taken from different studies, e.g. purified heart cells from two different studies, under the class “heart”. This allows us to use more than one signature for each cell-type, which increases the robustness of the algorithm in cases where the reference signatures are noisy. The algorithm first estimates 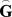 as if there are no classes. Then, all **W** columns associated with the same class are averaged and labeled according to that class.

### Microarray data

All microarray data was downloaded from GEO [14] as raw .CEL files and RMA normalized using R© package “affy”. The datasets and reference signatures used in each analysis were jointly quantile normalized using R© package “limma”. The following accession numbers were used for each dataset: **(1)** Liver-brain lung dataset [3] (GSE19830), with reference signatures of purified rat liver (GSE8252), brain (GSE3428), lung (GSE16849), intestine (GSE16849), heart (GSE5085) and granulosa (GSE13883) cells. All reference signatures were chosen from the same platform as the analyzed data - Affymetrix Rat Genome 230 2.0 Array. **(2)** Heart-brain dataset [15], with reference signatures of purified human myocardial (heart) cells from two different studies (GSE21610, GSE29819), brain cells from the entorhinal cortex (GSE4757) grey matter (GSE28146), oocytes (GSE12034) and hepatocyte (GSE31264). All reference signatures were from the same platform as the analyzed data - Human Genome U133 Plus 2.0 Array. **(3)** T-B-Monocytes dataset [4] (GSE11058), with reference signatures of purified T cell Jurkat (GSE7508, GSE30678), Monocyte THP-1 (GSE26868), B cell Raji (GSE12278, GSE13210) and IM-9 (GSE24147), IMC-1 NK (GSE19067) and MCF-10A epithelial (GSE10196) cell-lines. All reference signatures were from the same platform as the analyzed data - Affymetrix Human Genome U133 Plus 2.0 Array. **(4)** Prostate cancer dataset [7](GSE17951). The dataset included 154 patient samples with proportions of the tumor cells were available for 137 samples. Reference signatures included purified prostate tumor cell lines - DU145, PC3, CWR22Rv, LAPC4, C42B, LNCaP (GSE12348), benign prostate cells (GSE3325), normal prostate epithelial cells (GSE9951), stroma surrounding invasive prostate primary tumors and normal stroma (GSE26910). All reference signatures and analyzed data were from two similar platforms - Affymetrix Human Genome U133A Array and U133 Plus 2.0 Array.

### Mining purified signatures

To separate a heterogeneous tissue, the user should have some knowledge regarding the nature of the tissue that is being separated and its possible cell-type constituents. Purified signatures of the candidate cell-types may be found in public repositories such as GEO via a simple search for the required cell-type and species. The chosen signatures need not be from the same disease, tissue study or experiment as the heterogeneous tissue to be separated. In case there are many possible relevant options from different studies for a cell-type, one can input several signatures of the same cell-type to the algorithm and gather them under the same class. This was demonstrated in the heart-brain and T-B-Monocyte dataset examples. The limit in the total number of signatures used for all cell-types is the number of samples of the mixed tissue that is being separated, as explained under “Linear model for separation of gene-expression”. In case of uncertainty as to what cell-types constitute the tissue, one does not have to be precise and can over-guess by inputting many, even un-related, cell-types into the algorithm. Note that under-guessing the number of cell-types may cause ambiguities in the algorithm results, as explained above.

### Parameters setting

Parameters concerning majority voting (threshold, number of majority voting runs) and classes were set according to the nature of the cell-type signatures in each dataset, based on trial and error and common sense. In the case of majority voting, the more the input reference signatures are similar (such as in the T-B-Monocyte dataset [4], see also Figure S3A), the more likely that the algorithm will be farther away from the global minimum and therefore it will be harder to converge to a minimum that is close to the global minimum. Indeed, we noticed that the algorithm performs better with a lower threshold (i.e., a lower percentage of the number of times this cell-type is chosen out of the number of total runs) and a higher number of majority voting runs in such cases. In cases where the input reference signatures are less similar to each other (such as in the liver-brain-lung dataset [3], see also Figure S1A), less majority voting runs are needed to yield accurate results.

For classes' parameters, we observed that the algorithm encounters difficulties in separating cell-types for which the input reference signatures are very similar. In such instances, one might consider unifying these signatures under one class (where biologically relevant) or seek reference signatures from a different source. Observation of the input reference signatures, e.g. by drawing their heatmaps (Figures S1, S2, S3, S4A), can provide an indication regarding which reference signatures are similar.

The algorithm was run with the following parameters for each dataset: **(1)** liver-brain-lung dataset [3]: majority voting threshold = 70%, majority voting runs = 10, classes = none. **(2)** Heart-brain dataset [15]: majority voting threshold = 70%, majority voting runs = 10, classes = unifying the two brain and two heart cell types to the classes “brain” and “heart”, respectively. **(3)** T-B-Monocytes dataset [4]: majority voting threshold = 70%, majority voting runs = 20, classes = unifying the two B cell line types to the class “B cells”. **(4)** Prostate cancer dataset [7]: majority voting threshold = 70%, majority voting runs = 10, classes = unifying the 6 different prostate tumor cell lines to the class “tumor” and the epithelial and stromal cells to the class “other”.

## Supporting Information

Figure S1 **Blind separation of the liver-brain-lung dataset.** (**A**) Heatmap of the gene-expression signatures used in the liver-brain-lung dataset [3]. Top 10% variable probes (3,110) are shown. Publically available datasets mined from GEO were used for the signatures, as follows: liver - GSE8252, heart - GSE5085, granuloza - GSE13883, brain - GSE3428, lung - GSE16849, intestine - GSE16849. Gene expression from each dataset was averaged to yield a signature representative of that cell-type. Heatmap was generated in R© BioConductor using the gplots package. (**B**) Kullback-Leibler distances between the gene-expression of each separated cell type (CT1–CT3) to the gene-expression of each of the purified cell-types taken from the same study [3]. The distance is calculated between gene expression vectors; i.e. each vector represents a different cell-type and each entry of the vector represents the gene expression of a particular gene. The shortest distances between each separated cell-type and its corresponding purified cell-type are circled. (**C**) Kullback-Leibler distances between the known cell-type proportions and the estimated cell-type proportions (CT1–CT3) for all samples. The distance is calculated between vectors, such that each vector represents a different cell-type and each entry of the vector represents the relative proportion in a particular sample. The shortest distances between the estimated and known cell-type proportions are circled. (**D**) Kullback-Leibler distances between the purified gene-expression signatures taken from the same study [3], denoted as “real”, the estimated cell-type signatures inferred by the algorithm and the input cell-type reference signatures mined from GEO. The shortest distances are circled.
(TIF)

Figure S2 **Blind separation of the heart-brain dataset.** (**A**) Heatmap of the gene-expression signatures used in the heart-brain dataset [15]. Top 10% variable probes (5,468) are shown. Publically available datasets mined from GEO were used for the signatures, as follows: Brain cortex - GSE4757, Brain GM (grey matter) - GSE28146, ooctyes - GSE12034, hepatocytes - GSE31264, Heart 1 - GSE21610, Heart 2 - GSE29819. Gene expression from each dataset was averaged to yield a signature representative of that cell-type. Heatmap was generated in R© BioConductor using the gplots package. (**B**) Kullback-Leibler distances between the gene-expression of each separated cell type (CT1, CT2) to the gene-expression of each of the purified cell-types taken from the same study [15]. The distance is calculated between gene expression vectors; i.e. each vector represents a different cell-type and each entry of the vector represents the gene expression of a particular gene. The shortest distances between each separated cell-type and its corresponding purified cell-type are circled. (**C**) Kullback-Leibler distances between the known cell-type proportions and the estimated cell-type proportions (CT1, CT2) for all samples. The distance is calculated between vectors, such that each vector represents a different cell-type and each entry of the vector represents the relative proportion in a particular sample. The shortest distances between the estimated and known cell-type proportions are circled. (**D**) Kullback-Leibler distances between the purified gene-expression signatures taken from the same study [15], denoted as “real”, the estimated cell-type signatures inferred by the algorithm and the input reference cell-type signatures mined from GEO. The shortest distances are circled. The GEO accession numbers of the two signatures taken from different studies for both the heart and brain cell-types are denoted next to each comparison.
(TIF)

Figure S3 **Blind separation of the T-B-Monocytes dataset.** (**A**) Heatmap of the gene-expression signatures used in the T-B-Monocytes dataset [4]. Top 10% variable probes (2,734) are shown. Publically available datasets mined from GEO were used for the signatures, as follows: B IM9 cell line - GSE24147, B Raji cell line 1 - GSE12278, B Raji cell line 2 - GSE13210, Epithelial MCF10A cell line - GSE10196, Monocyte THP-1 cell-line - GSE26868, NK IMC-1 cell line - GSE19067, T Jurkat cell line 1 - GSE7508, T Jurkat cell line 2 - GSE30678. Gene expression from each dataset was averaged to yield a signature representative of that cell-type/dataset. Heatmap was generated in R© BioConductor using the gplots package. (**B**) Kullback-Leibler distances between the gene expressions of each separated cell-type (CT1–CT4) to the gene-expression of each of the purified cell-types taken from the same study^2^. The distance is calculated between gene expression vectors; i.e. each vector represents a different cell-type and each entry of the vector represents the gene expression of a particular gene. The shortest distances between each separated cell-type and its corresponding purified cell-type are circled. (**C**) Kullback-Leibler distances between the known cell-type proportions and the estimated cell-type proportions (CT1–CT4) for all samples. The distance is calculated between vectors, such that each vector represents a different cell-type and each entry of the vector represents the relative proportion in a particular sample. The shortest distances between the estimated and known cell-type proportions are circled. (**D**) Kullback-Leibler distances between the purified gene-expression signatures taken from the same study [4], denoted as “real”, the estimated cell-type signatures inferred by the algorithm and the input reference cell-type signatures mined from GEO. The shortest distances are circled. The GEO accession numbers of the two signatures taken from different studies for both the T (Jurkat) and B (Raji) cell lines are denoted next to each comparison. (**E**) Gene expression measurements of each separated cell-type (the two B cell-types were unified under the same class – “B cells”) plotted against the gene expression of its corresponding purified cell type from the same study. The known purified gene expression of the two B cell-types was averaged. (**F**) Kullback-Leibler distances between the gene expressions of each separated cell-type (CT1–CT3; the two B cell-types were unified under the same class – “B cells”) to the gene-expression of each of the purified cell-types taken from the same study^2^. The known purified gene expression of the two B cell-types was averaged. The distance is calculated between gene expression vectors; i.e. each vector represents a different cell-type and each entry of the vector represents the gene expression of a particular gene. The shortest distances between each separated cell-type and its corresponding purified cell-type are circled. (**G**) Kullback-Leibler distances between the known cell-type proportions and the estimated cell-type proportions (CT1–CT3) for all samples. The unified B cell-types (“B cells”) were compared to the sum of the proportions of the two B cell-types. The distance is calculated between vectors, such that each vector represents a different cell-type and each entry of the vector represents the relative proportion in a particular sample. The shortest distances between the estimated and known cell-type proportions are circled. (**H**) Comparisons between the known cell-type proportions and the estimated proportions per-sample for cell-types in the controlled datasets. Known proportions are shown in the grey columns, denoted by “s” and were measured as triplicates; estimates yielded by the blind separation algorithm are shown in the white columns and are denoted by “e”. (**I**) Known cell-type proportions plotted against the estimated cell-type proportions for all samples. The unified B cell-types (“B cells”) were compared to the sum of the proportions of the two B cell-types. Correlation coefficients between the known and estimated proportions are denoted for each cell-type.
(TIF)

Figure S4 **Blind separation of the prostate tumor dataset.** (**A**) Heatmap of the gene-expression signatures used in the heart-brain dataset [7]. Top 10% variable probes (2,228) are shown. Publically available datasets mined from GEO were used for the signatures, as follows: normal prostate epithelial cells (epithelial) - GSE9951, benign prostate tissue (benign) - GSE3325, stroma surrounding invasive prostate tumors (stromal surround) and normal stroma (stromal normal) - GSE26910, 6 prostate tumor cell-lines (DU145, PC3, CWR22Rv, LAPC4, C42B, LNCaP) - GSE12348. Gene expression from each dataset was averaged to yield a signature representative of that cell-type. Heatmap was generated in R© BioConductor using the gplots package. (**B**) Kullback-Leibler distances between the known cell-type proportions and the estimated cell-type proportions (CT1, CT2) for all samples. The distance is calculated between vectors, such that each vector represents a different cell-type and each entry of the vector represents the relative proportion in a particular sample. The shortest distances between the estimated and known cell-type proportions are circled. (**C**) Comparison of the known and estimated cell-type proportions per sample in the prostate cancer dataset. The average absolute error per sample is 12.44±12.41. Highlighted cells show the samples in which the absolute error was lower or equal to the average absolute error.
(TIF)

Figure S5 **Algorithm without the cell-type determination step – liver brain lung dataset.** A modified version of the separation algorithm without the cell-type determination step was run on the three cell-types liver-brain-lung dataset, using six, five and four reference cell-type signatures mined from GEO. The results show that in the case of algorithms that do not have a cell-type determination mechanism, such an over-fit (addition of extra cell-types) is insignificant if the resulting proportions of the additional cell-types are close to zero. However, this example clearly shows that this is not the case and that the over-fit significantly degraded the performance of the algorithm. Hence the cell-type determination step is crucial. (**A**) A run using all six input cell-types mined from GEO (liver, brain, lung, heart, intestine, granulosa). Correlations were calculated between the gene-expression of each separated cell type (CT1–CT6) to the gene-expression of each of the purified cell-types taken from the same study [3]. As the cell-type determination step was not performed, correlations between the purified signatures from the same study and the resulting cell-types were used to determine the identity of the resulting cell-types. The highest correlation between each separated cell-type and its corresponding purified cell-type are circled, thus pointing to the correct cell-types, i.e., liver = CT1, brain = CT3, lung = CT5. (**B**) Estimated cell-type proportions for all cell-types. The average absolute error per sample for cell-types CT1, CT3 and CT5 is 26.5±7.8. This is a much higher error compared to the error produced by our complete algorithm, which was 3.4%±2.3 (Figure 3A). Cells highlighted in orange show the real proportions (where liver = CT1, brain = CT3, lung = CT5); cells highlighted in grey are the cell-types which were mistakenly assumed to be present but were not removed because the cell-type determination step was not included here, as in our complete algorithm. (**C**) Correlations between the gene-expression of five input cell-types - liver, brain, lung, intestine and heart (CT1–CT5) and the gene-expression of each of the purified cell-types taken from the same study [3]. As the cell-type determination step was not performed, correlations between the purified signatures from the same study and the resulting cell-types were used to determine the identity of the resulting cell-types. The highest correlation between each separated cell-type and its corresponding purified cell-type are circled, pointing to the correct cell-types, i.e., liver = CT1, brain = CT2, lung = CT4. (**D**) Estimated cell-type proportions for five cell-types. The average absolute error per sample for cell-types CT1,CT2 and CT4 is 22.3±11.9. This is a much higher error compared to the error produced by our complete algorithm, which was 3.4%±2.3 (Figure 3A). Cells highlighted in orange show the real proportions (where liver = CT1, brain = CT2, lung = CT4); cells highlighted in grey are the cell-types which were mistakenly assumed to be present but were not removed because the cell-type determination step was not included here, as in our complete algorithm. (**E**) Correlations between the gene-expression of four input cell-types - liver, brain, lung and intestine (CT1–CT4) and the gene-expression of each of the purified cell-types taken from the same study [3]. As the cell-type determination step was not performed, correlations between the purified signatures from the same study and the resulting cell-types were used to determine the identity of the resulting cell-types. The highest correlation between each separated cell-type and its corresponding purified cell-type are circled, pointing to the correct cell-types, i.e., liver = CT1, brain = CT2, lung = CT3. (**F**) Estimated cell-type proportions for all cell-types. The average absolute error per sample for cell-types CT1,CT2 and CT3 is 11.7±4.75. This is a higher error compared to the error produced by our complete algorithm, which was 3.4%±2.3 (Figure 3A). Cells highlighted in orange show the real proportions (where liver = CT1, brain = CT2, lung = CT3); cells highlighted in grey are the cell-types which were mistakenly assumed to be present but were not removed because the cell-type determination step was not included here, as in our complete algorithm.
(TIF)

Figure S6 **Algorithm without the cell-type determination step – T-B-Monocytes dataset.** A modified version of the separation algorithm without the cell-type determination step was run on the four cell-types T-B-Monocytes dataset (which includes two different B cell line types), using six and five reference cell-type signatures mined from GEO. The results show that in the case of algorithms that do not have a cell-type determination mechanism, such an over-fit (addition of extra cell-types) is insignificant if the resulting proportions of the additional cell-types are close to zero. However, this example clearly shows that this is not the case and that the over-fit significantly degraded the performance of the algorithm. Hence the cell-type determination step is crucial. (**A**) A run using all six input cell-types mined from GEO (T-Jurkat, B-Raji, B-IM9, Monocytes, NK cells and epithelial cells). Correlations were calculated between the gene-expression of each separated cell type (CT1–CT6) to the gene-expression of each of the purified cell-types taken from the same study [4]. As the cell-type determination step was not performed, correlations between the purified signatures from the same study and the resulting cell-types were used to determine the identity of the resulting cell-types. The highest correlation between each separated cell-type and its corresponding purified cell-type are circled, pointing to the correct cell-types, i.e., T-Jurkat = CT1, B-Raji/IM9 = CT2, Monocytes = CT4. CT2 was identified as both B cell lines. (**B**) Estimated cell-type proportions for all cell-types. The average absolute error per sample for cell-types CT1,CT3 and CT4 is 21.2±6.7. This is a much higher error compared to the error produced by our complete algorithm, which was 5.7%±3.3 (Figure 3C). Cells highlighted in orange show the real proportions (where T-Jurkat = CT1, B-Raji/IM9 = CT2, Monocytes = CT4); cells highlighted in grey are the cell-types which were mistakenly assumed to be present but were not removed because the cell-type determination step was not included here, as in our complete algorithm. (**C**) Correlations between the gene-expression of five input cell-types – T-Jurkat, B-Raji, B-IM9, Monocytes and epithelial cells (CT1–CT5) and the gene-expression of each of the purified cell-types taken from the same study [4]. As the cell-type determination step was not performed, correlations between the purified signatures from the same study and the resulting cell-types were used to determine the identity of the resulting cell-types. The highest correlation between each separated cell-type and its corresponding purified cell-type are circled, pointing to the correct cell-types, i.e., T-Jurkat = CT1, B-Raji = CT2, B-IM9 = CT3, Monocytes = CT4. (**D**) Estimated cell-type proportions for all cell-types. The average absolute error per sample for cell-types CT1–CT4 is 13.7±4.9. This is a much higher error compared to the error produced by our complete algorithm, which was 5.7%±3.3 (Figure 3C). Cells highlighted in orange show the real proportions (where T-Jurkat = CT1, B-Raji = CT2, B-IM9 = CT3, Monocytes = CT4); cells highlighted in grey are the cell-types which were mistakenly assumed to be present but were not removed because the cell-type determination step was not included here, as in our complete algorithm.
(TIF)

Figure S7 **NNLS-based algorithm – liver brain lung dataset.** A NNLS (non-negative least squares)-based algorithm, which was used as a benchmark to most NNLS-based separation algorithms. The reference signatures were used to extract the proportions matrix. This algorithm was run on the three cell-types liver-brain-lung dataset, using six, five and four reference cell-type signatures mined from GEO. For NNLS-based algorithms which do not require any prior information, an over-fit (i.e., assume that there are more cell-types than actually exist) is insignificant if the resulting proportions of the additional cell-types are close to zero. However, this example clearly shows that this is not the case and that the over-fit significantly degraded the performance of the algorithm. Hence the cell-type determination step and the usage of NNMF (non-negative matrix factorization) is crucial. (**A**) A run using all six input cell-types mined from GEO (liver, brain, lung, heart, intestine, granulosa). Correlations were calculated between the gene-expression of each separated cell type (CT1–CT6) to the gene-expression of each of the purified cell-types taken from the same study [3]. As the cell-type determination step was not performed, correlations between the purified signatures from the same study and the resulting cell-types were used to determine the identity of the resulting cell-types. The highest correlation between each separated cell-type and its corresponding purified cell-type are circled, pointing to the correct cell-types, i.e., liver = CT1, brain = CT2, lung = CT4. (**B**) Estimated cell-type proportions for all cell-types. The average absolute error per sample for cell-types CT1,CT2 and CT4 is 24.2±8.72. This is a much higher error compared to the error produced by our complete algorithm, which was 3.4%±2.3 (Figure 3A). Cells highlighted in orange show the real proportions (where liver = CT1, brain = CT2, lung = CT4); cells highlighted in grey are the cell-types which were mistakenly assumed to be present but were not removed because the cell-type determination step was not included here, as in our complete algorithm. (**C**) Correlations between the gene-expression of five input cell-types - liver, brain, lung, intestine and heart (CT1–CT5) and the gene-expression of each of the purified cell-types taken from the same study [3]. As the cell-type determination step was not performed, correlations between the purified signatures from the same study and the resulting cell-types were used to determine the identity of the resulting cell-types. The highest correlation between each separated cell-type and its corresponding purified cell-type are circled, pointing to the correct cell-types, i.e., liver = CT1, brain = CT2, lung = CT4. (**D**) Estimated cell-type proportions for all cell-types. The average absolute error per sample for cell-types CT1,CT2 and CT4 is 18.4±5.81. This is a much higher error compared to the error produced by our complete algorithm, which was 3.4%±2.3 (Figure 3A). Cells highlighted in orange show the real proportions (where liver = CT1, brain = CT2, lung = CT4); cells highlighted in grey are the cell-types which were mistakenly assumed to be present but were not removed because the cell-type determination step was not included here, as in our complete algorithm. (**E**) Correlations between the gene-expression of four input cell-types - liver, brain, lung and intestine (CT1–CT4) and the gene-expression of each of the purified cell-types taken from the same study [3]. As the cell-type determination step was not performed, correlations between the purified signatures from the same study and the resulting cell-types were used to determine the identity of the resulting cell-types. The highest correlation between each separated cell-type and its corresponding purified cell-type are circled, pointing to the correct cell-types, i.e., liver = CT1, brain = CT2, lung = CT4. (**F**) Estimated cell-type proportions for all cell-types. The average absolute error per sample for cell-types CT1,CT2 and CT4 is 12.3±4.78. This is a higher error compared to the error produced by our complete algorithm, which was 3.4%±2.3 (Figure 3A). Cells highlighted in orange show the real proportions (where liver = CT1, brain = CT2, lung = CT4); cells highlighted in grey are the cell-types which were mistakenly assumed to be present but were not removed because the cell-type determination step was not included here, as in our complete algorithm.
(TIF)

Figure S8 **NNLS-based algorithm – T-B-Monocytes dataset.** A NNLS (non-negative least squares)-based algorithm, which was used as a benchmark to most NNLS-based separation algorithms. The reference signatures were used to extract the proportions matrix. This algorithm was run on the four cell-types T-B-Monocytes dataset (which includes two different B cell line types), using six and five reference cell-type signatures mined from GEO. For NNLS-based algorithms that do not require any prior information, an over-fit (i.e., assume that there are more cell-types than actually exist) is insignificant if the resulting proportions of the additional cell-types are close to zero. However, this example clearly shows that this is not the case and that the over-fit significantly degraded the performance of the algorithm. Hence the cell-type determination step and the usage of NNMF (non-negative matrix factorization) is crucial. (**A**) A run using six input cell-type signatures mined from GEO (T-Jurkat, B-Raji, B-IM9, Monocytes, NK cells and epithelial cells). Correlations were calculated between the gene-expression of each separated cell type (CT1–CT6) to the gene-expression of each of the purified cell-types taken from the same study [4]. As the cell-type determination step was not performed, correlations between the purified signatures from the same study and the resulting cell-types were used to determine the identity of the resulting cell-types. The highest correlation between each separated cell-type and its corresponding purified cell-type are circled, pointing to the correct cell-types, i.e., T-Jurkat = CT1, B-Raji/IM9 = CT2, Monocytes = CT4. CT2 was identified as both B cell lines. (**B**) Estimated cell-type proportions for all cell-types. The average absolute error per sample for cell-types CT1,CT2 and CT4 is 14.2±3.8. This is a much higher error compared to the error produced by our complete algorithm, which was 5.7%±3.3 (Figure 3C). Cells highlighted in orange show the real proportions (where T-Jurkat = CT1, B-Raji/IM9 = CT2, Monocytes = CT4); cells highlighted in grey are the cell-types which were mistakenly assumed to be present but were not removed because the cell-type determination step was not included here, as in our complete algorithm. (**C**) Correlations between the separated cell-types (CT1–CT5) and the gene-expression of each of the purified cell-types taken from the same study [4]. As the cell-type determination step was not performed, correlations between the purified signatures from the same study and the resulting cell-types were used to determine the identity of the resulting cell-types. The highest correlation between each separated cell-type and its corresponding purified cell-type are circled, pointing to the correct cell-types, i.e., T-Jurkat = CT1, B-Raji/IM9 = CT2, Monocytes = CT4. (**D**) Estimated cell-type proportions for all cell-types. The average absolute error per sample for cell-types CT1,CT2 and CT4 is 11.5±3.4. This is a much higher error compared to the error produced by our complete algorithm, which was 5.7%±3.3 (Figure 3C). Cells highlighted in orange show the real proportions (where T-Jurkat = CT1, B-Raji/IM9 = CT2, Monocytes = CT4); cells highlighted in grey are the cell-types which were mistakenly assumed to be present but were not removed because the cell-type determination step was not included here, as in our complete algorithm.
(TIF)

Text S1 **Algorithm pseudo code.** A matlab program of this code will be provided by the authors upon request.
(DOCX)

## References

[1] Cleator SJ, Powles TJ, Dexter T, Fulford L, Mackay A, et al (2006) The effect of the stromal component of breast tumours on prediction of clinical outcome using gene expression microarray analysis. Breast Cancer Res 8: R32. 16790077 10.1186/bcr1506 PMC1557729

[2] Mills JC, Roth KA, Cagan RL, Gordon JI (2001) DNA microarrays and beyond: completing the journey from tissue to cell. Nat Cell Biol 3: E175–178. 11483971 10.1038/35087108

[3] Shen-Orr SS, Tibshirani R, Khatri P, Bodian DL, Staedtler F, et al (2010) Cell type-specific gene expression differences in complex tissues. Nat Methods 7: 287–289. 20208531 10.1038/nmeth.1439 PMC3699332

[4] Abbas AR, Wolslegel K, Seshasayee D, Modrusan Z, Clark HF (2009) Deconvolution of blood microarray data identifies cellular activation patterns in systemic lupus erythematosus. PLoS One 4: e6098. 19568420 10.1371/journal.pone.0006098 PMC2699551

[5] Stuart RO, Wachsman W, Berry CC, Wang-Rodriguez J, Wasserman L, et al (2004) In silico dissection of cell-type-associated patterns of gene expression in prostate cancer. Proc Natl Acad Sci U S A 101: 615–620. 14722351 10.1073/pnas.2536479100 PMC327196

[6] Wang M, Master SR, Chodosh LA (2006) Computational expression deconvolution in a complex mammalian organ. BMC Bioinformatics 7: 328. 16817968 10.1186/1471-2105-7-328 PMC1559723

[7] Wang Y, Xia XQ, Jia Z, Sawyers A, Yao H, et al (2010) In silico estimates of tissue components in surgical samples based on expression profiling data. Cancer Res 70: 6448–6455. 20663908 10.1158/0008-5472.CAN-10-0021 PMC4411177

[8] Lu P, Nakorchevskiy A, Marcotte EM (2003) Expression deconvolution: a reinterpretation of DNA microarray data reveals dynamic changes in cell populations. Proc Natl Acad Sci U S A 100: 10370–10375. 12934019 10.1073/pnas.1832361100 PMC193568

[9] Venet D, Pecasse F, Maenhaut C, Bersini H (2001) Separation of samples into their constituents using gene expression data. Bioinformatics 17 Suppl 1: S279–287. 11473019 10.1093/bioinformatics/17.suppl_1.s279

[10] Erkkila T, Lehmusvaara S, Ruusuvuori P, Visakorpi T, Shmulevich I, et al (2010) Probabilistic analysis of gene expression measurements from heterogeneous tissues. Bioinformatics 26: 2571–2577. 20631160 10.1093/bioinformatics/btq406 PMC2951082

[11] Kuhn A, Thu D, Waldvogel HJ, Faull RL, Luthi-Carter R (2011) Population-specific expression analysis (PSEA) reveals molecular changes in diseased brain. Nat Methods 8: 945–947. 21983921 10.1038/nmeth.1710

[12] Lahdesmaki H, Shmulevich L, Dunmire V, Yli-Harja O, Zhang W (2005) In silico microdissection of microarray data from heterogeneous cell populations. BMC Bioinformatics 6: 54. 15766384 10.1186/1471-2105-6-54 PMC1274251

[13] Piper J, Pauca VP, Plemmons RJ, Giffin M (2004) Object Characterization from spectral data using nonnegative factorization and information theory. In In Proc. Amos Technical Conf., Maui, HI.

[14] Barrett T, Troup DB, Wilhite SE, Ledoux P, Rudnev D, et al (2009) NCBI GEO: archive for high-throughput functional genomic data. Nucleic Acids Res 37: D885–890. 18940857 10.1093/nar/gkn764 PMC2686538

[15] Affymetrix. Gene 1.0 ST Array Data Set. Available: http://www.affymetrix.com/support/technical/sample_data/gene_1_0_array_data.affx.

[16] Repsilber D, Kern S, Telaar A, Walzl G, Black GF, et al (2010) Biomarker discovery in heterogeneous tissue samples -taking the in-silico deconfounding approach. BMC Bioinformatics 11: 27. 20070912 10.1186/1471-2105-11-27 PMC3098067

