# Supplementary figures and images for: A Self-Directed Method for Cell-Type Identification and Separation of Gene Expression Microarrays
Source: PLoS Comput Biol. 2013 Aug 22;9(8):e1003189. doi: 10.1371/journal.pcbi.1003189 (PMC3749952; doi:10.1371/journal.pcbi.1003189)

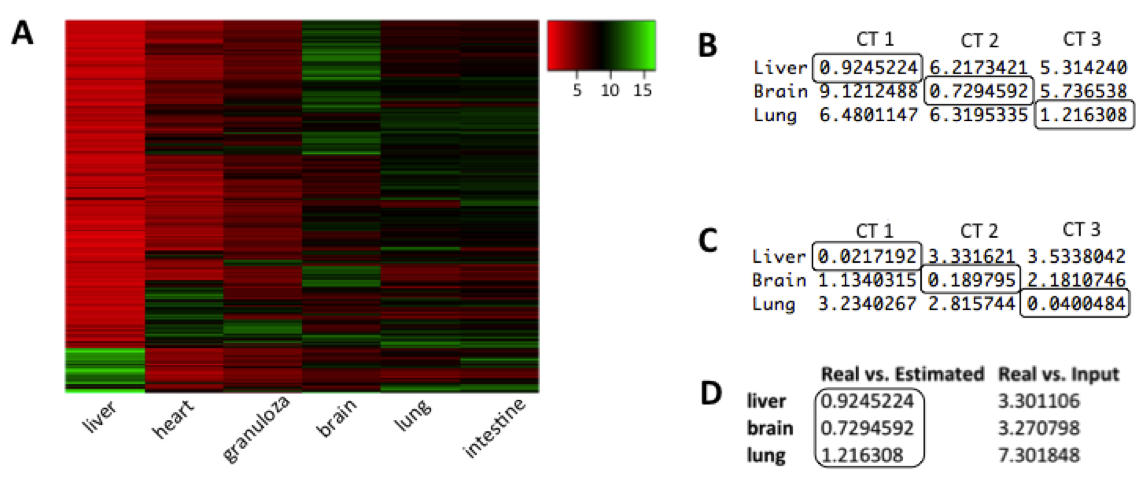

Supplement: Figure S1 — Blind separation of the liver-brain-lung dataset. (A) Heatmap of the gene-expression signatures used in the liver-brain-lung dataset [3]. Top 10% variable probes (3,110) are shown. Publically available datasets mined from GEO were used for the signatures, as follows: liver - GSE8252, heart - GSE5085, granuloza - GSE13883, brain - GSE3428, lung - GSE16849, intestine - GSE16849. Gene expression from each dataset was averaged to yield a signature representative of that cell-type. Heatmap was generated in R© BioConductor using the gplots package. (B) Kullback-Leibler distances between the gene-expression of each separated cell type (CT1–CT3) to the gene-expression of each of the purified cell-types taken from the same study [3]. The distance is calculated between gene expression vectors; i.e. each vector represents a different cell-type and each entry of the vector represents the gene expression of a particular gene. The shortest distances between each separated cell-type and its corresponding purified cell-type are circled. (C) Kullback-Leibler distances between the known cell-type proportions and the estimated cell-type proportions (CT1–CT3) for all samples. The distance is calculated between vectors, such that each vector represents a different cell-type and each entry of the vector represents the relative proportion in a particular sample. The shortest distances between the estimated and known cell-type proportions are circled. (D) Kullback-Leibler distances between the purified gene-expression signatures taken from the same study [3], denoted as “real”, the estimated cell-type signatures inferred by the algorithm and the input cell-type reference signatures mined from GEO. The shortest distances are circled. (TIF) [file pcbi.1003189.s001.tif]

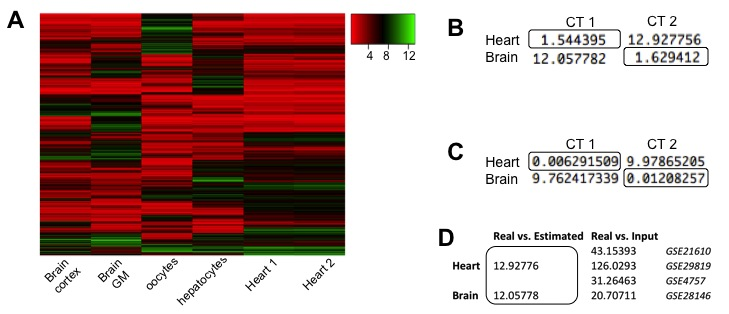

Supplement: Figure S2 — Blind separation of the heart-brain dataset. (A) Heatmap of the gene-expression signatures used in the heart-brain dataset [15]. Top 10% variable probes (5,468) are shown. Publically available datasets mined from GEO were used for the signatures, as follows: Brain cortex - GSE4757, Brain GM (grey matter) - GSE28146, ooctyes - GSE12034, hepatocytes - GSE31264, Heart 1 - GSE21610, Heart 2 - GSE29819. Gene expression from each dataset was averaged to yield a signature representative of that cell-type. Heatmap was generated in R© BioConductor using the gplots package. (B) Kullback-Leibler distances between the gene-expression of each separated cell type (CT1, CT2) to the gene-expression of each of the purified cell-types taken from the same study [15]. The distance is calculated between gene expression vectors; i.e. each vector represents a different cell-type and each entry of the vector represents the gene expression of a particular gene. The shortest distances between each separated cell-type and its corresponding purified cell-type are circled. (C) Kullback-Leibler distances between the known cell-type proportions and the estimated cell-type proportions (CT1, CT2) for all samples. The distance is calculated between vectors, such that each vector represents a different cell-type and each entry of the vector represents the relative proportion in a particular sample. The shortest distances between the estimated and known cell-type proportions are circled. (D) Kullback-Leibler distances between the purified gene-expression signatures taken from the same study [15], denoted as “real”, the estimated cell-type signatures inferred by the algorithm and the input reference cell-type signatures mined from GEO. The shortest distances are circled. The GEO accession numbers of the two signatures taken from different studies for both the heart and brain cell-types are denoted next to each comparison. (TIF) [file pcbi.1003189.s002.tif]

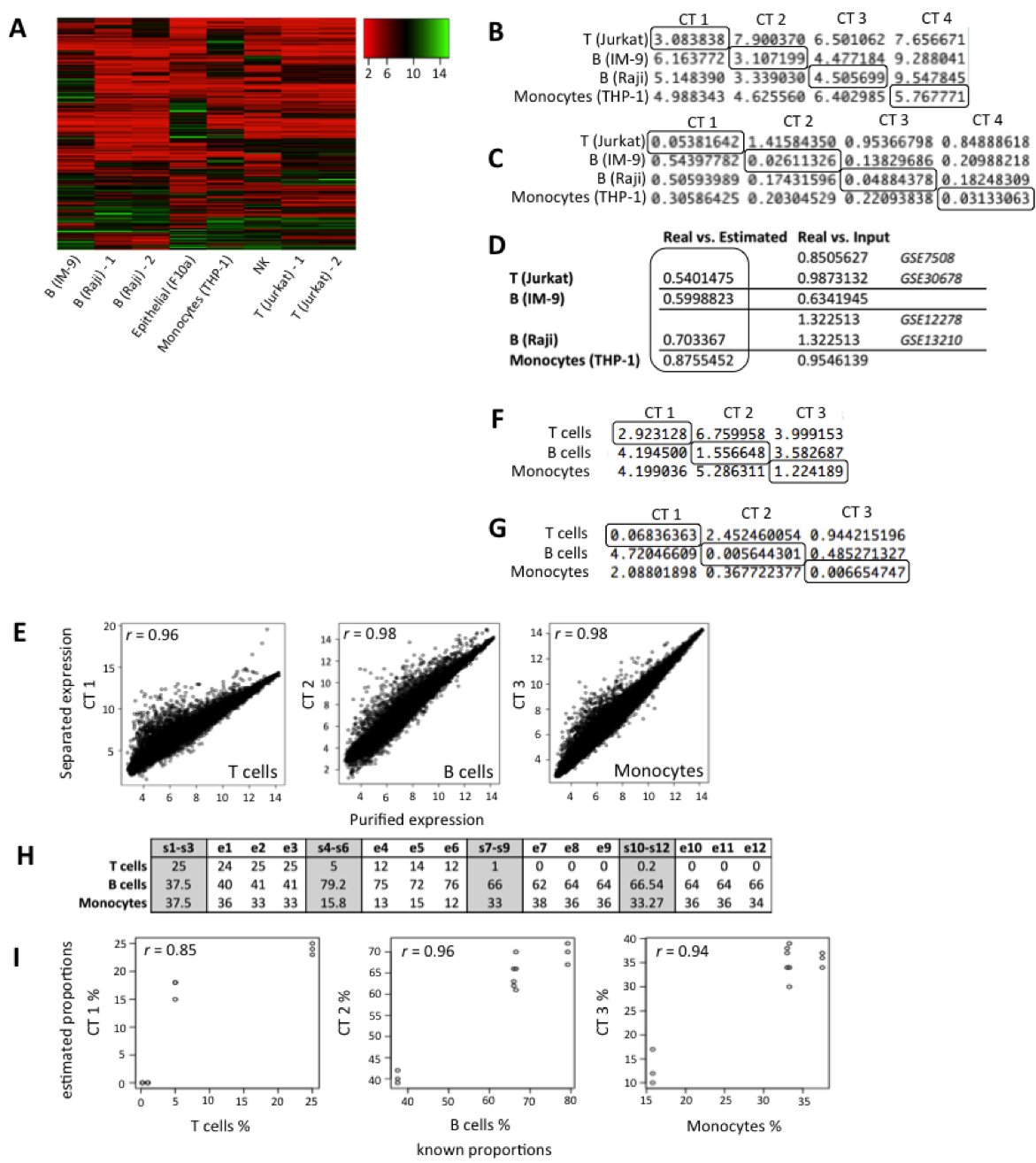

Supplement: Figure S3 — Blind separation of the T-B-Monocytes dataset. (A) Heatmap of the gene-expression signatures used in the T-B-Monocytes dataset [4]. Top 10% variable probes (2,734) are shown. Publically available datasets mined from GEO were used for the signatures, as follows: B IM9 cell line - GSE24147, B Raji cell line 1 - GSE12278, B Raji cell line 2 - GSE13210, Epithelial MCF10A cell line - GSE10196, Monocyte THP-1 cell-line - GSE26868, NK IMC-1 cell line - GSE19067, T Jurkat cell line 1 - GSE7508, T Jurkat cell line 2 - GSE30678. Gene expression from each dataset was averaged to yield a signature representative of that cell-type/dataset. Heatmap was generated in R© BioConductor using the gplots package. (B) Kullback-Leibler distances between the gene expressions of each separated cell-type (CT1–CT4) to the gene-expression of each of the purified cell-types taken from the same study2. The distance is calculated between gene expression vectors; i.e. each vector represents a different cell-type and each entry of the vector represents the gene expression of a particular gene. The shortest distances between each separated cell-type and its corresponding purified cell-type are circled. (C) Kullback-Leibler distances between the known cell-type proportions and the estimated cell-type proportions (CT1–CT4) for all samples. The distance is calculated between vectors, such that each vector represents a different cell-type and each entry of the vector represents the relative proportion in a particular sample. The shortest distances between the estimated and known cell-type proportions are circled. (D) Kullback-Leibler distances between the purified gene-expression signatures taken from the same study [4], denoted as “real”, the estimated cell-type signatures inferred by the algorithm and the input reference cell-type signatures mined from GEO. The shortest distances are circled. The GEO accession numbers of the two signatures taken from different studies for both the T (Jurkat) and B (Ra [file pcbi.1003189.s003.tif]

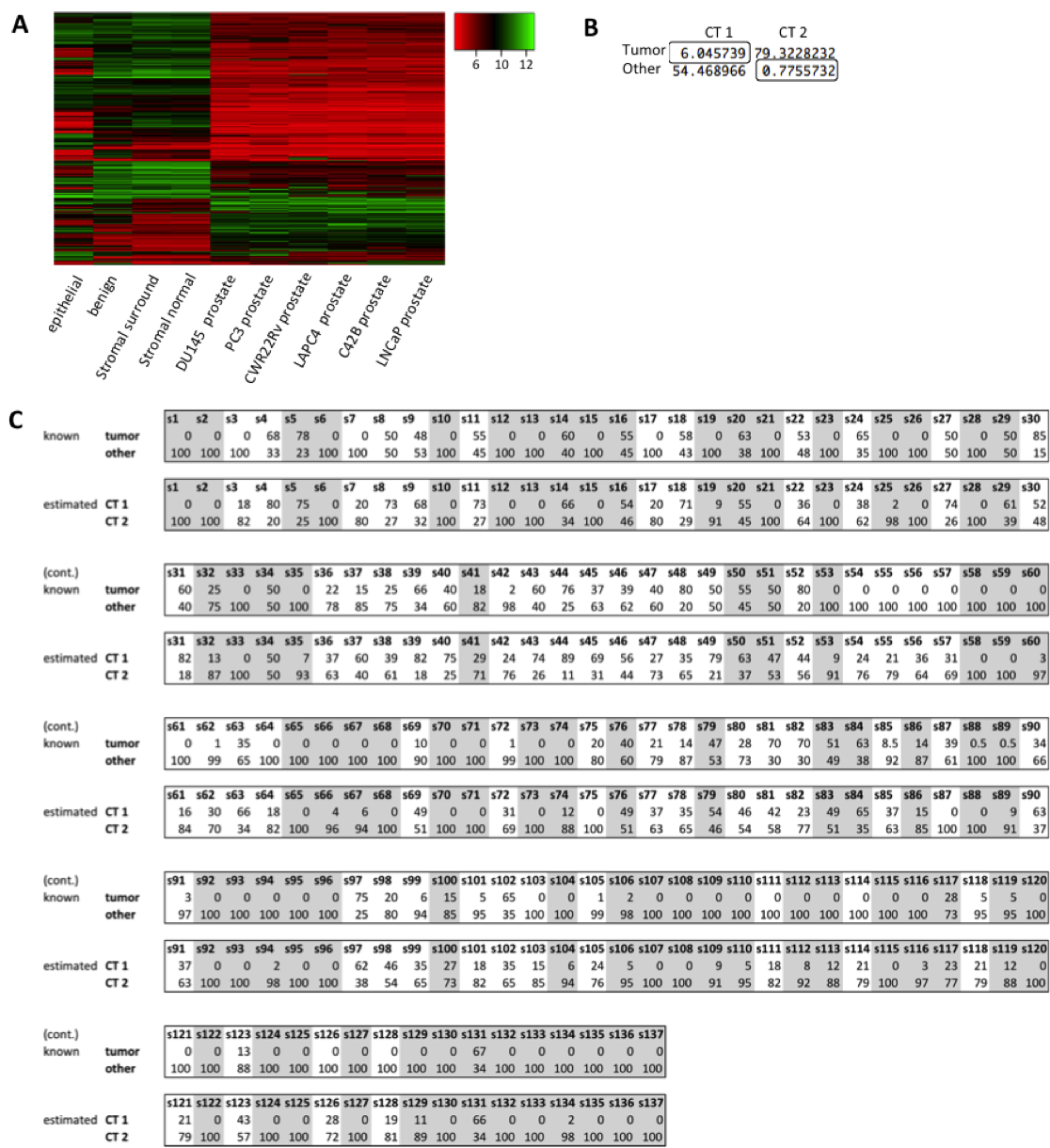

Supplement: Figure S4 — Blind separation of the prostate tumor dataset. (A) Heatmap of the gene-expression signatures used in the heart-brain dataset [7]. Top 10% variable probes (2,228) are shown. Publically available datasets mined from GEO were used for the signatures, as follows: normal prostate epithelial cells (epithelial) - GSE9951, benign prostate tissue (benign) - GSE3325, stroma surrounding invasive prostate tumors (stromal surround) and normal stroma (stromal normal) - GSE26910, 6 prostate tumor cell-lines (DU145, PC3, CWR22Rv, LAPC4, C42B, LNCaP) - GSE12348. Gene expression from each dataset was averaged to yield a signature representative of that cell-type. Heatmap was generated in R© BioConductor using the gplots package. (B) Kullback-Leibler distances between the known cell-type proportions and the estimated cell-type proportions (CT1, CT2) for all samples. The distance is calculated between vectors, such that each vector represents a different cell-type and each entry of the vector represents the relative proportion in a particular sample. The shortest distances between the estimated and known cell-type proportions are circled. (C) Comparison of the known and estimated cell-type proportions per sample in the prostate cancer dataset. The average absolute error per sample is 12.44±12.41. Highlighted cells show the samples in which the absolute error was lower or equal to the average absolute error. (TIF) [file pcbi.1003189.s004.tif]

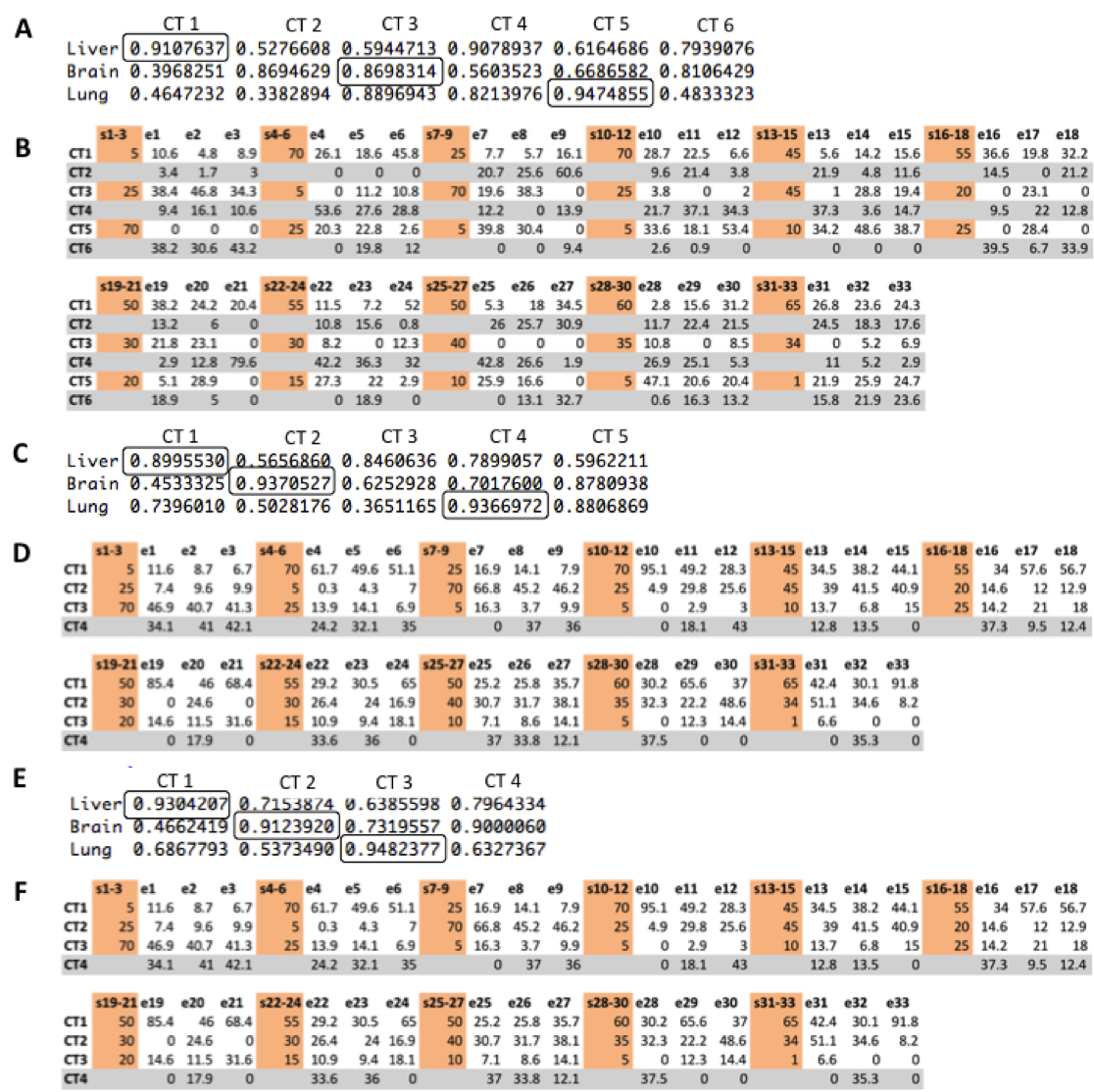

Supplement: Figure S5 — Algorithm without the cell-type determination step – liver brain lung dataset. A modified version of the separation algorithm without the cell-type determination step was run on the three cell-types liver-brain-lung dataset, using six, five and four reference cell-type signatures mined from GEO. The results show that in the case of algorithms that do not have a cell-type determination mechanism, such an over-fit (addition of extra cell-types) is insignificant if the resulting proportions of the additional cell-types are close to zero. However, this example clearly shows that this is not the case and that the over-fit significantly degraded the performance of the algorithm. Hence the cell-type determination step is crucial. (A) A run using all six input cell-types mined from GEO (liver, brain, lung, heart, intestine, granulosa). Correlations were calculated between the gene-expression of each separated cell type (CT1–CT6) to the gene-expression of each of the purified cell-types taken from the same study [3]. As the cell-type determination step was not performed, correlations between the purified signatures from the same study and the resulting cell-types were used to determine the identity of the resulting cell-types. The highest correlation between each separated cell-type and its corresponding purified cell-type are circled, thus pointing to the correct cell-types, i.e., liver = CT1, brain = CT3, lung = CT5. (B) Estimated cell-type proportions for all cell-types. The average absolute error per sample for cell-types CT1, CT3 and CT5 is 26.5±7.8. This is a much higher error compared to the error produced by our complete algorithm, which was 3.4%±2.3 (Figure 3A). Cells highlighted in orange show the real proportions (where liver = CT1, brain = CT3, lung = CT5); cells highlighted in grey are the cell-types which were mistakenly assumed to be present but were not removed because the cell-type determination step was not included here, as in our complete algorithm. (C) C [file pcbi.1003189.s005.tif]

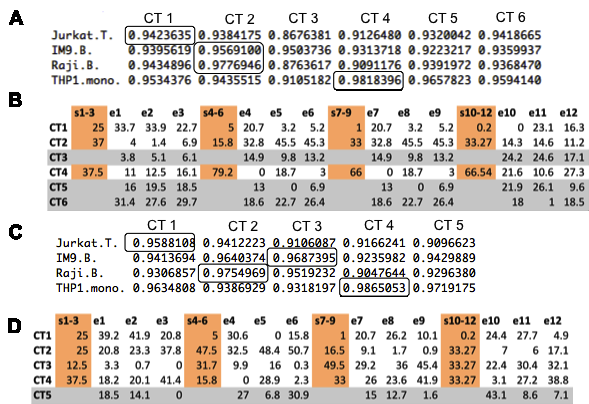

Supplement: Figure S6 — Algorithm without the cell-type determination step – T-B-Monocytes dataset. A modified version of the separation algorithm without the cell-type determination step was run on the four cell-types T-B-Monocytes dataset (which includes two different B cell line types), using six and five reference cell-type signatures mined from GEO. The results show that in the case of algorithms that do not have a cell-type determination mechanism, such an over-fit (addition of extra cell-types) is insignificant if the resulting proportions of the additional cell-types are close to zero. However, this example clearly shows that this is not the case and that the over-fit significantly degraded the performance of the algorithm. Hence the cell-type determination step is crucial. (A) A run using all six input cell-types mined from GEO (T-Jurkat, B-Raji, B-IM9, Monocytes, NK cells and epithelial cells). Correlations were calculated between the gene-expression of each separated cell type (CT1–CT6) to the gene-expression of each of the purified cell-types taken from the same study [4]. As the cell-type determination step was not performed, correlations between the purified signatures from the same study and the resulting cell-types were used to determine the identity of the resulting cell-types. The highest correlation between each separated cell-type and its corresponding purified cell-type are circled, pointing to the correct cell-types, i.e., T-Jurkat = CT1, B-Raji/IM9 = CT2, Monocytes = CT4. CT2 was identified as both B cell lines. (B) Estimated cell-type proportions for all cell-types. The average absolute error per sample for cell-types CT1,CT3 and CT4 is 21.2±6.7. This is a much higher error compared to the error produced by our complete algorithm, which was 5.7%±3.3 (Figure 3C). Cells highlighted in orange show the real proportions (where T-Jurkat = CT1, B-Raji/IM9 = CT2, Monocytes = CT4); cells highlighted in grey are the cell-types which were mistakenly assumed to be present but w [file pcbi.1003189.s006.tif]

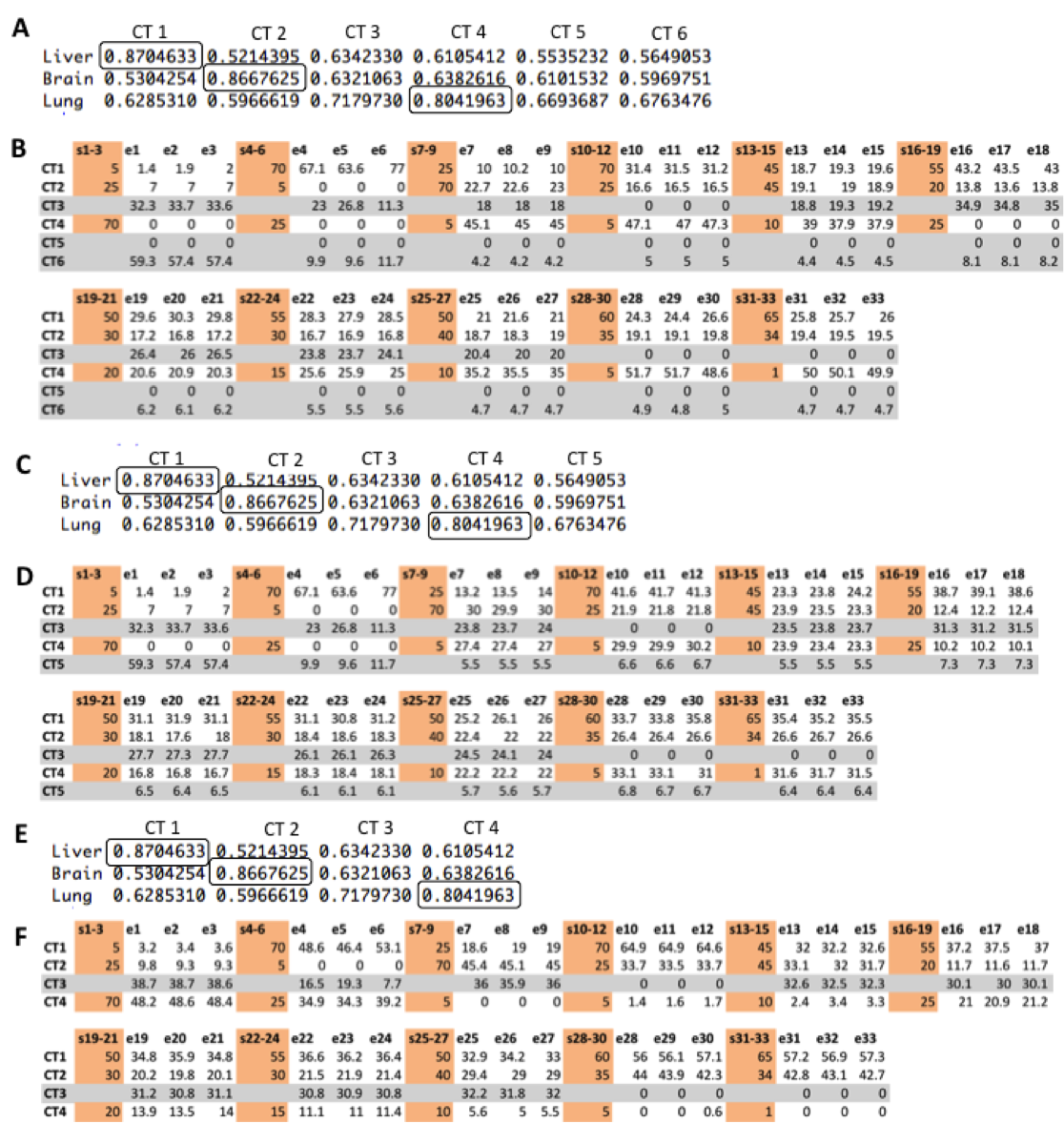

Supplement: Figure S7 — NNLS-based algorithm – liver brain lung dataset. A NNLS (non-negative least squares)-based algorithm, which was used as a benchmark to most NNLS-based separation algorithms. The reference signatures were used to extract the proportions matrix. This algorithm was run on the three cell-types liver-brain-lung dataset, using six, five and four reference cell-type signatures mined from GEO. For NNLS-based algorithms which do not require any prior information, an over-fit (i.e., assume that there are more cell-types than actually exist) is insignificant if the resulting proportions of the additional cell-types are close to zero. However, this example clearly shows that this is not the case and that the over-fit significantly degraded the performance of the algorithm. Hence the cell-type determination step and the usage of NNMF (non-negative matrix factorization) is crucial. (A) A run using all six input cell-types mined from GEO (liver, brain, lung, heart, intestine, granulosa). Correlations were calculated between the gene-expression of each separated cell type (CT1–CT6) to the gene-expression of each of the purified cell-types taken from the same study [3]. As the cell-type determination step was not performed, correlations between the purified signatures from the same study and the resulting cell-types were used to determine the identity of the resulting cell-types. The highest correlation between each separated cell-type and its corresponding purified cell-type are circled, pointing to the correct cell-types, i.e., liver = CT1, brain = CT2, lung = CT4. (B) Estimated cell-type proportions for all cell-types. The average absolute error per sample for cell-types CT1,CT2 and CT4 is 24.2±8.72. This is a much higher error compared to the error produced by our complete algorithm, which was 3.4%±2.3 (Figure 3A). Cells highlighted in orange show the real proportions (where liver = CT1, brain = CT2, lung = CT4); cells highlighted in grey are the cell-types which were mistakenly [file pcbi.1003189.s007.tif]

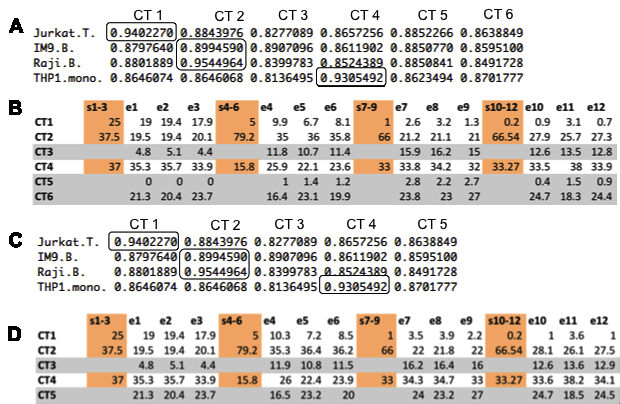

Supplement: Figure S8 — NNLS-based algorithm – T-B-Monocytes dataset. A NNLS (non-negative least squares)-based algorithm, which was used as a benchmark to most NNLS-based separation algorithms. The reference signatures were used to extract the proportions matrix. This algorithm was run on the four cell-types T-B-Monocytes dataset (which includes two different B cell line types), using six and five reference cell-type signatures mined from GEO. For NNLS-based algorithms that do not require any prior information, an over-fit (i.e., assume that there are more cell-types than actually exist) is insignificant if the resulting proportions of the additional cell-types are close to zero. However, this example clearly shows that this is not the case and that the over-fit significantly degraded the performance of the algorithm. Hence the cell-type determination step and the usage of NNMF (non-negative matrix factorization) is crucial. (A) A run using six input cell-type signatures mined from GEO (T-Jurkat, B-Raji, B-IM9, Monocytes, NK cells and epithelial cells). Correlations were calculated between the gene-expression of each separated cell type (CT1–CT6) to the gene-expression of each of the purified cell-types taken from the same study [4]. As the cell-type determination step was not performed, correlations between the purified signatures from the same study and the resulting cell-types were used to determine the identity of the resulting cell-types. The highest correlation between each separated cell-type and its corresponding purified cell-type are circled, pointing to the correct cell-types, i.e., T-Jurkat = CT1, B-Raji/IM9 = CT2, Monocytes = CT4. CT2 was identified as both B cell lines. (B) Estimated cell-type proportions for all cell-types. The average absolute error per sample for cell-types CT1,CT2 and CT4 is 14.2±3.8. This is a much higher error compared to the error produced by our complete algorithm, which was 5.7%±3.3 (Figure 3C). Cells highlighted in orange show the real proportions [file pcbi.1003189.s008.tif]
