# Supplementary material for: A Self-Directed Method for Cell-Type Identification and Separation of Gene Expression Microarrays
Source: PLoS Comput Biol. 2013 Aug 22;9(8):e1003189. doi: 10.1371/journal.pcbi.1003189 (PMC3749952; doi:10.1371/journal.pcbi.1003189)
Supplement: Text S1 — Algorithm pseudo code. A matlab program of this code will be provided by the authors upon request. (DOCX) [file pcbi.1003189.s009.docx]

**Text S1**

**Algorithm Pseudo code**

A matlab program of this code will be provided by the authors upon request.

**Input: (A)** $m x n$ mixed matrix $\mathbf{M}$ and $m x k_{max}$ reference signatures matrix $\mathbf{L}$ with non-negative entries, where $m$ is the number of genes, $n$ is the number of samples and $k_{max}$ is the number of initial cell-types, **(B)** A collection of $T^{'}=\left\{ T_{1}^{'}\ldots T_{k_{max}}^{'} \right\}$ cell-type labels where $T_{k}^{'}$ is the label of the $k^{th}$ column in $\mathbf{L}$, **(C)** number of majority voting runs $Q$.

**For** $v=1\ldots Q$

(1) Initialize: $h_{kj}$ with non-negative values $1\leq k\leq k_{max}$, $1\leq j\leq n$

Initialize: $w_{ik}(v)$ with $l_{ik}$ $1\leq i\leq m$, $1\leq k\leq k_{max}$

Scale columns of $\mathbf{W}(v)$ to sum to one.

(2) Get $\mathbf{W}(v)$ and $\mathbf{H}$ using NMF, as described in Piper et al. ^12^:

$H_{kj}^{(t)}=H_{kj}^{(t-1)}\frac{\left[ \left( \mathbf{W}^{(t-1)} \right)^{T}\mathbf{M} \right]_{kj}}{\left[ \left( \mathbf{W}^{(t-1)} \right)^{T}\mathbf{W}^{(t-1)}\mathbf{H}^{\boldsymbol{(}t-1\boldsymbol{)}} \right]_{kj}+\epsilon}$

$W_{ik}^{(t)}=H_{ik}^{(t-1)}\frac{\left[ {\mathbf{M}\left( \mathbf{H}^{(t)} \right)}^{T} \right]_{ik}}{\left[ \mathbf{W}^{(t-1)}\mathbf{H}^{(t)}\left( \mathbf{H}^{(t)} \right)^{T} \right]_{ik}+\epsilon}$

where $\epsilon\approx{10}^{-9}$ is used to avoid possible division by zero and $t$ refers to
 the NMF iteration

(3) Determine $\hat{k}_{CT}$(*v*) according to (4)

(4) Determine $\hat{\mathbf{G}}$(*v*) and its cell-type labels as the chosen columns in $\mathbf{W(}v\mathbf{)}$

(5) Set $Z_{k}\left( v \right)=1$ if the label $T_{k}^{'}$ is chosen, $Z_{k}\left( v \right)=0$ otherwise

**End For**

(6) Determine the final cell-type identities:

$\hat{T}=\left\{ \hat{T}_{1}, \ldots, \hat{T}_{k_{CT}} \right\}$, where $T_{k}^{'}\in\hat{T}$ if $\frac{1}{Q}\sum_{v=1}^{Q} Z_{k}(v)\geq threshold$

(7) Determine the final $\hat{\mathbf{G}}$: for $i=1,\ldots,\hat{k}_{CT}\left( v \right)$

$$\hat{g}_{i}=\mathrm{average}\left\{ \hat{g}_{k}\left( v \right), v=1,\ldots,Q, such that a label of \hat{g}_{k}\left( v \right)\mathrm{is}\hat{T}_{i} \right\}$$

(8) Set $\hat{k}_{CT}$ to the number of columns in the matrix $\hat{\mathbf{G}}$

(9) Determine $\hat{\mathbf{C}}$ according to Eq. (5)

**Output:** $\hat{\mathbf{G}}$, $\hat{\mathbf{C}}$, $\hat{k}_{CT}$

* To use classes the algorithm requires the following sets $\Psi_{p}, p=1\ldots P$, where $P$ is the number of classes and each $\Psi_{p}$ contains the collection of labels $T_{k}^{'}$ that are affiliated with class $p$. Note that there may be classes that contain a single label. Let$\psi_{p}$ be the label of class $p$, then the algoritm outputs $\hat{\psi}=\left\{ \hat{\psi}_{1}, \ldots, \hat{\psi}_{k_{CT}} \right\}$ which is the estimated labels of classes as follows:

Change (6) such that:

$\hat{\psi}=\left\{ \hat{\psi}_{1}, \ldots, \hat{\psi}_{k_{CT}} \right\}$, where $\psi_{p}\in\hat{T}$ if $\frac{1}{Q}\sum_{k:T_{k}^{'}\in C_{p}} \sum_{v=1}^{Q} Z_{k}(v)\geq threshold$

Change (7) to:

Determine the final $\hat{\mathbf{G}}$: for $i=1,\ldots,\hat{k}_{CT}\left( v \right)$

$\hat{g}_{i}=\mathrm{average}\left\{ \hat{g}_{k}\left( v \right), v=1,\ldots,Q, such that a label of \hat{g}_{k}\left( v \right) is in \hat{\psi}_{i} \right\}$
